# A Comprehensive Molecular Phylogeny of Dalytyphloplanida (Platyhelminthes: Rhabdocoela) Reveals Multiple Escapes from the Marine Environment and Origins of Symbiotic Relationships

**DOI:** 10.1371/journal.pone.0059917

**Published:** 2013-03-25

**Authors:** Niels Van Steenkiste, Bart Tessens, Wim Willems, Thierry Backeljau, Ulf Jondelius, Tom Artois

**Affiliations:** 1 Centre for Environmental Sciences, Hasselt University, Diepenbeek, Belgium; 2 Department of Biology, University of Antwerp, Antwerp, Belgium; 3 Department of Invertebrates and Joint Experimental Molecular Unit, Royal Belgian Institute of Natural Sciences, Brussels, Belgium; 4 Department of Invertebrate Zoology, Swedish Museum of Natural History, Stockholm, Sweden; Consiglio Nazionale delle Ricerche (CNR), Italy

## Abstract

In this study we elaborate the phylogeny of Dalytyphloplanida based on complete 18S rDNA (156 sequences) and partial 28S rDNA (125 sequences), using a Maximum Likelihood and a Bayesian Inference approach, in order to investigate the origin of a limnic or limnoterrestrial and of a symbiotic lifestyle in this large group of rhabditophoran flatworms. The results of our phylogenetic analyses and ancestral state reconstructions indicate that dalytyphloplanids have their origin in the marine environment and that there was one highly successful invasion of the freshwater environment, leading to a large radiation of limnic and limnoterrestrial dalytyphloplanids. This monophyletic freshwater clade, Limnotyphloplanida, comprises the taxa Dalyelliidae, Temnocephalida, and most Typhloplanidae. Temnocephalida can be considered ectosymbiotic Dalyelliidae as they are embedded within this group. Secondary returns to brackish water and marine environments occurred relatively frequently in several dalyeliid and typhloplanid taxa. Our phylogenies also show that, apart from the Limnotyphloplanida, there have been only few independent invasions of the limnic environment, and apparently these were not followed by spectacular speciation events. The distinct phylogenetic positions of the symbiotic taxa also suggest multiple origins of commensal and parasitic life strategies within Dalytyphloplanida. The previously established higher-level dalytyphloplanid clades are confirmed in our topologies, but many of the traditional families are not monophyletic. Alternative hypothesis testing constraining the monophyly of these families in the topologies and using the approximately unbiased test, also statistically rejects their monophyly.

## Introduction

**Figure 1:**
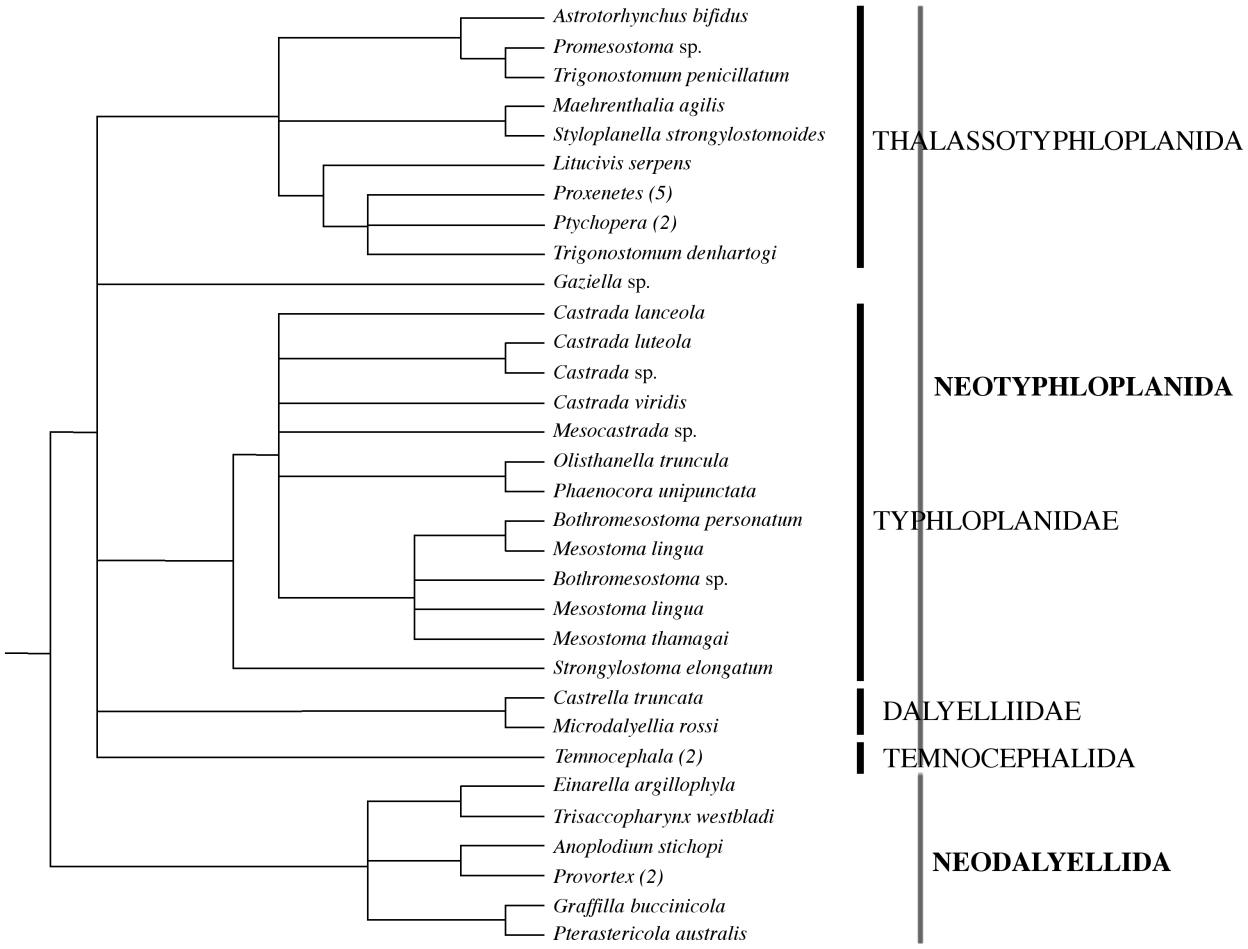
Dalytyphloplanid phylogeny redrawn after Willems et al. [**1**]. Dalytyphloplanida consists of Neotyphloplanida and Neodalyellida. Numbers of species per terminal taxon are given in parentheses if >1.

With about 1530 described species, Rhabdocoela is one of the most species-rich taxa of non-neodermatan flatworms. Classical taxonomy, mainly based on the presence of a proboscis and the pharynx morphology, recognises four large groups within Rhabdocoela: Kalyptorhynchia (with a frontal proboscis and a pharynx rosulatus, i.e. a mostly globular pharynx with a muscular septum and a more or less vertical axis), Typhloplanoida (with pharynx rosulatus, without proboscis), Dalyellioida (with pharynx doliiformis, i.e. a barrel-shaped, anteriorly-situated pharynx with a muscular septum and a horizontal axis) and Temnocephalida (with a pharynx doliiformis and ectosymbiotic on freshwater invertebrates and chelonians). Willems et al. [1] were the first to thoroughly and intentionally explore rhabdocoel phylogenetic relationships using 18S rDNA sequences. In contrast to the traditional morphology-based phylogenies, this study divided Rhabdocoela into two large clades: Kalyptorhynchia (±530 species) and Dalytyphloplanida (±1000 species). Dalytyphloplanids are found worldwide in marine, brackish water, limnic and limnoterrestrial environments. It comprises the Typhloplanoida, Dalyellioida and Temnocephalida. Table 1 summarizes the traditional classification of the families and subfamilies in the Dalytyphloplanida. Yet, Willems et al. [1] showed that both the “Typhloplanoida” and “Dalyellioida” are polyphyletic and their representatives are scattered all over the dalytyphloplanid tree (Fig. 1). Consequently, the clades and names established by Willems et al. [1] will be followed here. As such, Dalytyphloplanida is divided into Neodalyellida and Neotyphloplanida, the former roughly consisting of all marine “Dalyellioida”, while the latter being further divided into the well-supported Thalassotyphloplanida and a poorly-supported, trichotomous freshwater clade comprising Typhloplanidae, Dalyelliidae and Temnocephalida [1] (Fig. 1). The monophyly of this freshwater clade was already suggested by Jondelius and Thollesson [2], though needed further support because it was based on methodologically ill-founded conclusions [3]. Later on, Willems et al. [1] re-addressed the monophyly of this clade, but could not definitively resolve the issue because of too limited taxon sampling. Hence, the existence of the freshwater clade in the Neotyphloplanida needs to be explored further using more representatives than Willems et al. [1] did. Addressing the monophyly of the neotyphloplanid freshwater clade is interesting because it would show whether or not the Dalytyphloplanida follow a pattern of repeated independent invasions of freshwater habitats by marine ancestors, as observed in other major clades of Platyhelminthes (e.g. Catenulida, Macrostomida, Prorhynchida, Continenticola, Kalyptorhynchia; see [4]).

**Table 1:** Traditional classification of the taxa comprising Dalytyphloplanida.

Typhloplanoida Bresslau, 1933	Dalyellioida Bresslau, 1933
**Promesostomidae Den Hartog, 1964**	**Dalyelliidae Graff, 1908**
Adenorhynchinae Ax and Heller, 1970	**Provorticidae Beklemischew, 1927**
Brinkmanniellinae Luther, 1948	Neokirgellinae Oswald et al., 2010
Promesostominae Luther, 1948	Provorticinae Luther, 1962
*Gaziella* De Clerck and Schockaert, 1995	Haplovejdovskyinae Luther, 1962
*Paraproboscifer* De Clerck, 1994	*Eldenia reducta* Ax, 2008
*Vauclusia* Willems et al., 2004	Hypoblepharinidae Böhmig, 1914
**Trigonostomidae Graff, 1905**	**Luridae Sterrer and Rieger, 1990**
Mariplanellinae Ax and Heller, 1970	**Graffillidae Graff, 1908**
Paramesostominae Luther, 1948	Bresslauillinae Bresslau, 1933
Trigonostominae Luther, 1948	Pseudograffillinae Meixner, 1938
**Typhloplanidae Graff, 1905**	Graffillinae Graff, 1905
Ascophorinae Findenegg, 1924	**Pterastericolidae Meixner, 1926**
Cephalopharynginae Hochberg, 2004	**Umagillidae Wahl, 1910**
Mesophaenocorinae Noreña et al., 2006	Bicladinae Stunkard and Corliss, 1950
Mesostominae Bresslau, 1933	Collastominae Wahl, 1910
Olisthanellinae Bresslau, 1933	Umagillinae Wahl, 1910
Opistominae Luther, 1963	
Phaenocorinae Wahl, 1910	**Temnocephalida Bresslauand Reisinger, 1933**
Protoplanellinae Reisinger, 1924	
Rhynchomesostominae Bresslau, 1933	**Temnocephalidae Monticelli, 1899**
Typhloplaninae Bresslau, 1933	**Diceratocephalidae Joffe et al., 1998**
**Byrsophlebidae Graff, 1905**	**Didymorchidae Bresslau and Reisinger, 1933**
**Kytorhynchidae Rieger, 1974**	**Actinodactylellidae Benham, 1901**
**Solenopharyngidae Graff, 1882**	**Scutariellidae Annandale, 1912**
Anthopharynginae Ehlers, 1972	
Lenopharynginae Ehlers, 1972	
Solenopharynginae Ehlers, 1972	
**Carcharodopharyngidae Bresslau, 1933**	
**Ciliopharyngiellidae Ax, 1952**	

Hundreds of species of non-neodermatan flatworms in 35 families live in symbiosis with other organisms, ranging from commensalism to obligate parasitism. With ±250 species, the majority of these symbionts are dalytyphloplanids: Temnocephalida, Umagillidae, Pterastericolidae and Graffillidae. A few species of rhabdocoel symbionts were included in the analysis of Willems et al. [1], showing that they are not closely related to each other. This raised questions on the origins of commensalism and parasitism in dalytyphloplanids. Moreover, it would be interesting to further explore the relationships of the symbiotic rhabdocoels.

Against this background, the present work aims at extending and deepening the work of Willems et al. [1] in order to: (a) establish a sound molecular phylogeny of the Dalytyphloplanida using more taxa and gene fragments, (b) explore the transition from the marine to the freshwater environment in the Dalytyphloplanida, and (c) corroborate the phylogenetic positions of symbiotic taxa and their implications for the evolution of commensalism and parasitism in free-living flatworms.

## Materials and Methods

### 1. Collection and selection of taxa

From 2007 to 2011, 157 dalytyphloplanid taxa were collected worldwide for DNA sequence analysis of 18S and 28S rDNA. They represent 10 of the 13 traditional families, 17 of the 24 traditional subfamilies and 57 of the 168 genera of free-living taxa. In addition, sequences of 11 symbiotic taxa were also included (4 Temnocephalidae, 2 Graffillidae, 2 Pterastericolidae and 3 Umagillidae). This taxon sampling involved 81 marine and 76 freshwater taxa, yet many marine and some limnic species are also found in brackish water, while five “limnic” species are actually limnoterrestrial. All necessary permits were obtained for the described field studies. Permission for sampling in protected areas was granted by the governing authorities (Doñana Biological Station, Doñana National Park, Spain; Ezemvelo KZN Wildlife, iSimangaliso, South Africa; Museum and Art Gallery of the Northern Territory, NT, Australia). No specific permits were required for the described field studies in other locations, which were not privately owned or protected. The field studies did not involve the collection of endangered or protected species.

Marine and some brackish water animals were collected from sediment and algae using the MgCl_2_ decantation method, while the oxygen depletion method was deployed to obtain limnic and brackish water specimens from aquatic vegetation, mud and organic material [5]. Limnoterrestrial animals were extracted from mosses and forest soils with the Baermann pan method [6]. Specimens were identified by studying live animals, whole mounts and stained serial sections. Additional specimens were fixed in 95% ethanol and stored at −20°C until DNA extraction. Specimen collection and sequence data and GenBank accession numbers are provided in Table S1. Dalytyphloplanid sequences from previous studies [1], [7]–[9] were taken from GenBank and are also listed in Table S1 with their GenBank accession numbers.

Some specimens could not be identified to genus or species level because either there was not enough material available or they were new to science and not yet formally described. Nevertheless we include these taxa in our analyses in order to recover as much phylogenetic information as possible. The results of Willems et al. [1] showed that Kalyptorhynchia is the sister group of the Dalytyphloplanida. Therefore *Acrorhynchides robustus* (Polycystididae) and *Placorhynchus octaculeatus* (Placorhynchidae), the only two kalyptorhynch species for which both 18S rDNA and 28S rDNA sequences are available, were chosen as outgroup.

### 2. DNA extraction and amplification

Genomic DNA was extracted from entire specimens using the QIAamp® DNA Micro Kit with QIAamp MinElute® columns (QIAGEN) and following the manufacturer's protocol. Extracts were stored in duplicate (40 and 20 µl) for each specimen.

For most specimens complete 18S rDNA (±1780–1800 bp) and partial 28S rDNA (±1600–1700 bp) gene fragments were amplified using the primers and PCR protocols listed in Tables 2 and S1. Most rDNA fragments were easily amplified using the standard primer pairs TimA/TimB for the 18S rDNA gene [9] and LSU5/LSUD6.3 for the partial 28S rDNA gene [10] and their respective standardised PCR protocols. Nevertheless, for a relatively large number of taxa PCR conditions had to be optimised. This could involve nothing more than increasing the number of PCR cycles to developing new primers (with often a reduction of the number of amplifiable bp as a consequence). In particular, specimens of *Promesostoma*, *Kaitalugia*, some Trigonostomidae, Solenopharyngidae and many marine Dalyellioida could only be amplified with taxon-specific primer pairs (Tables 2 and S1). Amplicons yielded ±1700 bp for Prom18SFb/Prom18SRb, ±1640 bp for Neodal18SF/Neodal18SR, ±1300 bp for Neodal28SFa/Neodal28SRb and ±1600 bp for SolenoF1/SolenoR.

**Table 2:** Primers and usage.

Primers & Regime	Direction	Primer sequence (5′–3′)	Usage	Reference
**18S/SSU Primers**				
TimA	Forward	AMCTGGTTGATCCTGCCAG	PCR/Sequencing	[82]
TimB	Reverse	AGGTGAACCTGCAGATGGATCA	PCR/Sequencing	[82]
S30	Forward	GCTTGTCTCAAAGATTAAGCC	Nested PCR	[82]
5FK	Reverse	TTCTTGGCAAATGCTTTCGC	Nested PCR/Sequencing	[82]
4FB	Forward	CCAGCAGCCGCGGTAATTCCAG	Nested PCR	[82]
1806R	Reverse	CCTTGTTACGACTTTTACTTCCTC	Nested PCR	[82]
PCR regime TimA/TimB	DK18S: 95°C at 5 min 10 s, 30×(94°C at 30 s, 55°C at 30 s, 72°C at 1 min 30 s), 72°C at 5 min			
	DK18S35cycli: 95°C at 5 min 10 s, 35×(94°C at 30 s, 55°C at 30 s, 72°C at 1 min 30 s), 72°C at 5 min			
Prom18SFb	Forward	ACGGTGAGACCGCGAATGGC	PCR/Sequencing	This study
Prom18SRb	Reverse	AACAAGGTTTCCGTAGGTGAACCTGC	PCR/Sequencing	This study
PCR regime Prom18SFb/Prom18SRb	DK18S65: 95°C at 5 min 10 s, 30×(94°C at 30 s, 65°C at 30 s, 72°C at 1 min 30 s), 72°C at 5 min			
Neodal18SF	Forward	TGGTTGATCCTGCCAGTAATGATATGC	PCR/Sequencing	This study
Neodal18SR	Reverse	CGCCCGTCGCTACTACCGATT	PCR/Sequencing	This study
PCR regime Neodal18SF/Neodal18SR	DK18S65: 95°C at 5 min 10 s, 30×(94°C at 30 s, 65°C at 30 s, 72°C at 1 min 30 s), 72°C at 5 min			
600F	Forward	GGTGCCAGCAGCCGCGGT	Sequencing	[1]
600R	Reverse	ACCGCGGCTGCTGGCACC	Sequencing	[1]
1100F	Forward	CAGAGGTTCGAAGACGATC	Sequencing	[82]
1100R	Reverse	GATCGTCTTCGAACCTCTG	Sequencing	[82]
18S7F	Forward	GCAATAACAGGTCTGTGATGC	Sequencing	[82]
18S7FK	Reverse	GCATCACAGACCTGTTATTGC	Sequencing	[82]
5F	Forward	GCGAAAGCATTTGCCAAGAA	Sequencing	[82]
7F	Forward	GCAATAACAGGTCTGTGATGC	Sequencing	[82]
7FK	Reverse	GCATCACAGACCTGTTATTGC	Sequencing	[82]
**28S/LSU Primers**				
LSU5	Forward	TAGGTCGACCCGCTGAAYTTA	PCR/Sequencing	[10]
LSUD6.3	Reverse	GACTGAAGTGGAGAAGGGTTCC	PCR/Sequencing	[10]
PCR regime LSU5/LSU6.3	DK28S: 95°C at 5 min 10 s, 30×(94°C at 1 min, 50°C at 1 min, 72°C at 1 min 30 s), 72°C at 5 min			
	DK28S35cycli: 95°C at 5 min 10 s, 35×(94°C at 1 min, 50°C at 1 min, 72°C at 1 min 30 s), 72°C at 5 min			
LSUD6.3B	Reverse	GAGAAGGGTTCCATGTGAACAGC	PCR/Sequencing	This study
PCR regime LSU5/LSU6.3B	DK28S: 95°C at 5 min 10 s, 30×(94°C at 1 min, 50°C at 1 min, 72°C at 1 min 30 s), 72°C at 5 min			
Neodal28SFa	Forward	ACGGCGAGTGAACAGGGAAAAGC	PCR/Sequencing	This study
Neodal28SRb	Reverse	AGACAGCAGGACGGTGGCCA	PCR/Sequencing	This study
PCR regime Neodal28SFa/Neodal28SRb	DK28S65: 95°C at 5 min 10 s, 30×(94°C at 1 min, 65°C at 1 min, 72°C at 1 min 30 s), 72°C at 5 min			
SolenoF1	Forward	CGGCGAGTGAACAGGAATTAGCCC	PCR/Sequencing	This study
SolenoR	Reverse	AGGCCGATGTGGAGAAGGGT	PCR/Sequencing	This study
PCR regime SolenoF1/SolenoR	DK28S68: 95°C at 5 min 10 s, 30×(94°C at 1 min, 68°C at 1 min, 72°C at 1 min 30 s), 72°C at 5 min			
L300F	Forward	CAAGTACCGTGAGGGAAAGTTG	Sequencing	[10]
L300R	Reverse	CAACTTTCCCTCACGGTACTTG	Sequencing	[10]
L1200F	Forward	CCCGAAAGATGGTGAACTATGC	Sequencing	[10]
L1200R	Reverse	GCATAGTTCACCATCTTTCGG	Sequencing	[10]
L1600F	Forward	GCAGGACGGTGGCCATGGAAG	Sequencing	[10]
L1600R	Reverse	CTTCCATGGCCACCGTCCTGC	Sequencing	[10]

Most PCR reactions were carried out using the 0.2 ml PuReTaq Ready-To-Go PCR beads (GE Healthcare). For each reaction 2.5 µl of each primer (5 µM), 3 µl of DNA and 17 µl of purified water were assembled in the RTG-PCR tubes to make up a volume of 25 µl. Some reactions were performed in 0.2 ml tubes using the HotStar*Taq®*Plus DNA polymerase (QIAGEN) kit. Each of these reactions contained 2.5 µl dNTPs (2 mM), 2.5 µl PCR buffer (10×) with 15 mM MgCl_2_, 2.5 µl of each primer (5 µM), 0.25 µl *Taq* DNA polymerase, 3 µl of DNA and 11.75 µl purified water. All PCR reactions were carried out in an Eppendorf Mastercycler gradient or a Techne TC-5000 thermocycler. PCR products (5 µl) were verified on a 1.4% agarose gel and stained with ethidium bromide or GelRed™. Some products were purified with the QIAquick® PCR Purification Kit (QIAGEN) following the manufacturer's instructions, but for most PCR products, purification was performed by Macrogen (Korea). In the rare cases when multiple bands occurred while checking the PCR product, the bands corresponding with the target fragment were excised and purified using the QIAquick® Gel Extraction Kit (QIAGEN) according to the manufacturer's protocol. Sequencing was done by Macrogen (Korea) using BigDye™ terminator cycling conditions. Reaction products were purified with ethanol precipitation and run with a 3730XL Automatic DNA Sequencer 3730XL.

### 3. Alignment and dataset

Before alignment, consensus sequences were assembled from contigs in Staden v1.6.0 [11] and subjected to a BLAST search [12] on the NCBI website (http://www.ncbi.nlm.nih.gov) to check for possible contaminations. Subsequently, sequences were aligned with the structural Q-INS-i algorithm in MAFFT [13]–[14]. Manual editing of the alignments was avoided by selecting and filtering ambiguous positions with the alignment masking program Aliscore using the settings described by Misof and Misof [15]. The 5′ and 3′ alignment ends of the 18S and 28S rDNA datasets were trimmed and subsequently both alignments were concatenated in Geneious Pro 5.3.4 (Biomatters Ltd). Processed (aligned and masked) sequences that were identical, were removed from the alignments before phylogenetic analysis (Table S2). Final alignments were tested for substitution saturation with DAMBE v5.2.57 according to Xia's method for more than 32 OTUs [16]. Proportions of invariant sites were obtained in jModeltest v0.1.1 [17] when testing the best fitting substitution model for our dataset. Saturation tests were performed on all sites with gaps treated as unknown data (Table S3).

### 4. Phylogenetic analyses

Maximum likelihood (ML) analyses were conducted separately for the three final datasets in RAxML v7.2.8 performing 100 independent runs of thorough searches and 1000 non-parametric bootstrap replicates under the GTR+CAT model [18]–[19]. This model of evolution is a more workable approximation for GTR+Γ and recommended by the program instead of GTR+ Γ+I. The latter was the most appropriate model of evolution for the concatenated and 28S dataset as selected in jModeltest 0.1.1 [17] by both the Akaike (AIC) and Bayesian (BIC) information criteria. Although the lnL value was better for the GTR+Γ+I model, AIC and BIC for the 18S alignment were slightly more in favour of the TIM2+Γ+I model. Gaps were treated as missing data. Bootstrap replicates were used to construct majority rule consensus trees and bootstrap support values (BS) were plotted on best-scoring trees in SumTrees v2.0.2 of the DendroPy package [20].

Bayesian inferences (BI) were done in MrBayes v3.1.2 [21] under the GTR+Γ+I model (nst = 6; rates = invgamma), using default prior settings, Metropolis coupled Markov chain Monte Carlo sampling with one cold and three heated chains (default; temp = 0.2) in two independent simultaneous runs for 10 million generations. Gaps were treated as missing data. Convergence was determined based on the logL values and the average standard deviation of split frequencies. Trees were sampled every 100^th^ generation after a burnin of 2,500,000 generations (burnin = 25,000). Majority rule consensus trees were constructed from the remaining 75,000 trees. All Bayesian analyses were performed on the Bioportal at Oslo University [22].

Figtree v1.3.1 [23] was used for tree drawing and displaying bootstrap support values (BS) and posterior probabilities (PP). Identical sequences removed pre-analysis were manually reinserted into the final phylogenetic trees. We consider BS and PP significant above a 0.70 and 0.95 threshold respectively, as was proposed by Alfaro et al. [24].

### 5. Hypothesis testing

To test alternative hypotheses on the monophyly of some dalytyphloplanid families, the ML searches in RAxML as described above (100 independent runs under the GTR+CAT model) were repeated with different constrained topologies. These constrained topologies were subsequently tested against the unconstrained ML topologies with the approximately unbiased (AU) test [25]. Site-wise log-likelihoods were calculated in Tree-Puzzle v5.2 [26] and processed in Consel v0.1i [27] to calculate *p*-values for the AU test.

### 6. Ancestral state reconstructions

To explore habitat shifts between marine/brackish water environments and limnic environments, we followed the procedure of Pagel et al. [28]. Using *Acrorhynchides robustus* as enforced outgroup MrBayes v3.1.2. was rerun on the concatenated dataset with settings and indications of convergence as in the phylogenetic analysis proper. MrBayes parameter files (.p) were then analysed using Tracer v1.5 [29] to obtain the LnL integrated autocorrelation time (IACT = 202) and effective sampling size (ESS = 371) [30]. After burnin (25,000), the posterior of 75,000 trees (.t) was consequently “thinned” with Burntrees v0.1.9 [31] by subsampling every 202^nd^ tree to approximate the ESS. A majority rule consensus tree was constructed from the resulting 372 trees in SumTrees v2.0.2 and compared to the original consensus tree to ensure that topology, branch lengths and posterior probabilities remained stable. *Acrorhynchides robustus* was pruned from all trees in the thinned tree file with Simmap v1.0 [32] because branch lengths between ingroup and outgroup have been shown to affect ancestral state reconstructions [33]–[34].

Ancestral states were reconstructed for 11 well-supported nodes (PP≥0.95) in the 18S+28S phylogeny in BayesTraits v1.0 [28]. The binary traits, “exclusively or predominantly marine/brackish water (M/B)” and “exclusively or predominantly limnic/limnoterrestrial (L/LT)” were treated as habitat states. Each of the analysed nodes represents the most recent common ancestor (MRCA) of a certain number of descendants from both marine/brackish water and freshwater habitats. MRCA-MCMC analyses were run for each node (AddMRCA) using a multistate model with two states. A reversible-jump hyperprior approach with an exponential prior seeded from a uniform distribution between 0 and 30 (rjhp 0 30) was applied to reduce uncertainty and arbitrariness of prior choice [28]. Trial runs allowed to estimate a value for the rate deviation parameter (rd 8) and to obtain an acceptance rate between 20–40%. MCMC chains were run for 50.1 million iterations (it 50,100,000) and sampled every 1000 iterations (sa 1000) after a burnin of 100,000 iterations (bi 100,000). Nodes were subsequently fossilised (fossil) to test whether one of the two habitat states is significantly more supported. For some analyses with fossil priors, rate deviations parameters were set to 15 (rd 15) to secure acceptance rates between 20–40%. Each analysis was repeated three times because the harmonic means of log-likelihoods can be unstable. Bayes factors were used as test statistics and calculated from the average harmonic means for the fossilised states [28].

## Results

The combined dataset initially consisted of 2890 bp for 159 sequences (incl. the outgroup); 18S and 28S datasets comprised 1614 bp for 156 sequences and 1276 bp for 125 sequences respectively. After removing identical sequences (Table S2) from the initial alignments, the numbers of sequences were 155, 145 and 121 for the combined, 18S and 28S alignments respectively. There were no indications of substitution saturation (Table S3).

**Figure 2:**
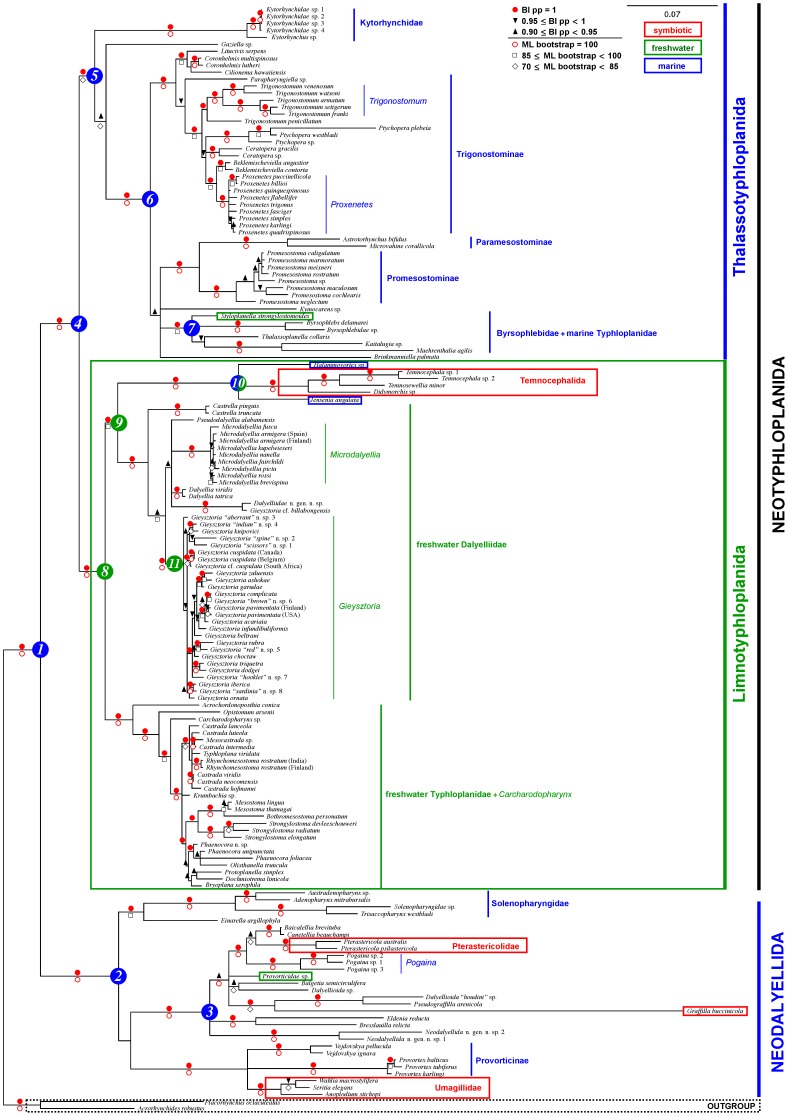
Majority-rule consensus tree from the Bayesian analysis of the concatenated 18S+28S rDNA dataset. Dalytyphloplanida consists of Neotyphloplanida and Neodalyellida. Symbols above the branches indicate bootstrap values from the ML analysis. No symbols indicate support values below the thresholds in the legend. Branches have been collapsed when both the posterior probabilities and bootstrap support values are below the thresholds in the legend. Scale bars represent numbers of substitutions/site. Node numbers correspond with the most recent common ancestors of the ancestral state reconstructions.

ML topologies were generally congruent with the BI phylogenies, which are shown in Figs. 2, S1 and S2. The concatenated data yielded better-resolved trees with overall higher support values than the single gene trees (see Figs. 2, S1 and S2). Dalytyphloplanida consists of two well-supported sister clades: Neodalyellida and Neotyphloplanida.

All trees provide strong support for three clades within Neodalyellida: (a) the *Einarella+*Solenopharyngidae clade, (b) the Provorticinae+Umagillidae clade and (c) a “mixed” clade including Graffillidae, Pterastericolidae, different provorticid genera (*Canetellia*, *Baicalellia*, *Pogaina*, *Balgetia*, *Eldenia*, *Provorticidae* sp.) and some undescribed taxa. Some of the traditional families, i.e. Solenopharyngidae, Pterastericolidae and Umagillidae, are monophyletic with high support, while Provorticidae and Graffillidae are polyphyletic.

Most ML and BI trees (Figs. 2 and S2) support the division of Neotyphloplanida in two large clades: (a) a marine clade, Thalassotyphloplanida s.l. consisting of Kytorhynchidae, Trigonostomidae, Promesostomidae (except for *Einarella argillophyla*), Byrsophlebidae and *Gaziella* sp. (Promesostomidae), and (b) a freshwater clade, for which we propose the new name Limnotyphloplanida, which is defined as the last common ancestor of the taxa Dalyelliidae, Temnocephalida, most Typhloplanidae, and all of the descendants of that common ancestor.

The 28S and 18S+28S (Figs. 2 and S2), but not the 18S trees (Fig. S1) support Kytorhynchidae as sister taxon to all other Thalassotyphloplanida s.s. The position of *Gaziella* sp. is somewhat doubtful, yet both the 18S and 18S+28S data (no 28S data available) suggest it to be the sister taxon of the remaining Thalassotyphloplanida s.s. The latter clade consists of a polytomy of several well-supported clades: (a) a clade with some Promesostomidae (*Litucivis*, *Coronhelmis*) and all Trigonostominae (Trigonostomidae), (b) a clade with Promesostominae (Promesostomidae) and Paramesostominae (Trigonostomidae), and (c) a clade with Byrsophlebidae and some peculiar Typhloplanidae (*Styloplanella strongylostomoides*, *Thalassoplanella collaris*, *Kaitalugia* sp.). The position of *Brinkmanniella palmata* and *Kymocarens* sp. (both Promesostomidae) in these clades is uncertain. Anyway, the traditional families Trigonostomidae, Promesostomidae and Byrsophlebidae are clearly not monophyletic. Conversely, most trees and support values support the monophyly of the thalassotyphloplanid genera represented by two or more species in this study: *Coronhelmis*, *Trigonostomum*, *Ptychopera*, *Ceratopera*, *Beklemischeviella*, *Proxenetes* and *Promesostoma*.

The monophyly of the freshwater clade, Limnotyphloplanida, is generally very well supported for all datasets (BS 98–100; PP 1.00) except for the 28S ML tree (BS 64). Within this freshwater clade, all topologies and support values (BS 98–100; PP 1.00) confirm the monophyly of three major clades: (a) a clade comprising Temnocephalida and the marine Dalyelliidae *Jensenia angulata* and *Halammovortex* sp., (b) a clade comprising all freshwater Dalyelliidae, and (c) a clade comprising nearly all Typhloplanidae and *Carcharodopharynx* sp. (Carcharodopharyngidae). ML and BI trees of the concatenated and 18S data point to a clade (BS 76–86; PP 0.99–1) grouping the Temnocephalida+*Jensenia+Halammovortex* clade and the other Dalyelliidae as the sister clade of the Typhloplanidae+*Carcharodopharynx* clade (Figs. 2 and S1). For 28S (Fig. S2), this clade is weakly supported (BS 60; PP 0.88). Within Dalyelliidae, all genera with two or more representatives in this study (*Microdalyellia*, *Dalyellia*, *Castrella* and all species of *Gieysztoria* except for *Gieysztoria* cf. *billabongensis*) are monophyletic with high support values (BS 93–100; PP 0.99–1), but their more detailed relationships remain partly unresolved. Only the position of *Gieysztoria* cf. *billabongensis*, which forms a separate clade with an unidentified species from India, jeopardises the monophyly of *Gieysztoria*. Relations within the Typhloplanidae+*Carcharodopharynx* clade are conflicting and often unresolved. Conversely, the positions of *Acrochordonoposthia conica*, *Opistomum arsenii* and *Carcharodopharynx* as hierarchically nested sister taxa relative to all other freshwater Typhloplanidae are well-supported in most topologies (except for the position of *O. arsenii* and *Carcharodopharynx* in the 28S trees). The remaining taxa in the freshwater Typhloplanidae are either unresolved, paraphyletic or receive various degrees of support.

The AU tests rejected the constrained trees forcing the monophyly of all “traditional” families that appear para- and polyphyletic in our trees (Table 3). Only the AU test of the 28S tree constrained by monophyly of Byrsophlebidae was not rejected, possibly because fewer taxa were used in this analysis. The results of the ancestral state reconstructions in the 18S+28S phylogeny are summarised in Table 4 and Fig. 2. Bayes factors clearly provide statistical support for the expected habitat state of the most recent common ancestor of the analysed clades, i.e. exclusively or predominantly marine/brackish water (M/B) for Dalytyphloplanida (node 1), Neodalyellida (node 2), Neotyphloplanida (node 4), Thalassotyphloplanida s.l. and s.s. (nodes 5 and 6), the Byrsophlebidae+“marine” Typhloplanidae clade (node 7) and the “mixed” neodalyellid clade (node 3); exclusively or predominantly limnic/limnoterrestrial (L/LT) for Limnotyphloplanida (node 8), Dalyelliidae s.l. (node 9) and *Gieysztoria* (node 11). Only for the Temnocephalida+*Jensenia+Halammovortex* clade (node 10), the support for the best ancestral state model is average (BF 1.64).

**Table 3:** *p*-values of the AU tests in Consel v0.1i.

Trees	18S+28S	18S	28S
Constrained monophyly Byrsophlebidae	0.002	4e-04	*0.738*
Optimal ML tree	0.998	1.000	*0.262*
Constrained monophyly Dalyelliidae	2e-06	1e-04	2e-96
Optimal ML tree	1.000	1.000	1.000
Constrained monophyly Promesostomidae	2e-10	5e-54	0.003
Optimal ML tree	1.000	1.000	0.997
Constrained monophyly Provorticidae	6e-07	0.006	2e-28
Optimal ML tree	1.000	0.994	1.000
Constrained monophyly Trigonostomidae	3e-05	6e-06	0.003
Optimal ML tree	1.000	1.000	0.997
Constrained monophyly Typhloplanidae	3e-67	3e-06	1e-05
Optimal ML tree	1.000	1.000	1.000
Constrained monophyly Brinkmanniellinae	3e-132	2e-05	0.065
Optimal ML tree	1.000	1.000	0.935

**Table 4:** Ancestral state reconstruction using BayesTraits v3.1.2.

Node (mrca)	Best model	BF	Support for best model
1	M/B	6,88	strong
2	M/B	12,67	very strong
3	M/B	12,70	very strong
4	M/B	7,17	strong
5	M/B	9,83	strong
6	M/B	12,63	very strong
7	M/B	11,38	very strong
8	L/LT	2,16	positive
9	L/LT	2,37	positive
10	M/B	1,64	average
11	L/LT	28,71	very strong

Habitat states are categorised as marine/brackish water (M/B) or limnic/limnoterrestrial (L/LT). Analysed nodes representing most recent common ancestors (MRCA) are visualised in Fig. 2. Bayes Factors (BF) were calculated with the harmonic means (HM) of the fossilised states: BF = 2*(HM_best model_−HM_worse model_). Support for the best model is described as “average” (BF>0), “positive” (BF>2), “strong” (BF>5) or “very strong” (BF>10).

## Discussion

With “only” 41 dalytyphloplanid 18S rDNA sequences, Willems et al. [1] established a phylogeny of Dalytyphloplanida that was very different from the traditional classification of rhabdocoels into “Typhloplanoida”, “Dalyellioida”, Temnocephalida and Kalyptorhynchia. These findings invoked important questions on the origin of the freshwater taxa and the phylogenetic position of symbiotic dalytyphloplanids. Our phylogenies and ancestral state reconstructions suggest that Dalytyphloplanida, Neotyphloplanida and Thalassotyphloplanida have (eury)haline origins and that apart from some smaller colonisation events in both Neodalyellida and Thalassotyphloplanida, one very successful invasion of the freshwater environment by a common ancestor of Dalyelliidae, Typhloplanidae and Temnocephalida took place. The analyses presented here are the first to specifically deal with these issues using DNA sequence data. In addition, they provide a wealth of new phylogenetic insights and taxonomic information.

### 1. Single escape from the marine environment?

#### 1.1. Major radiation after a single colonisation event

Except for the 28S ML topology (BS 64), the monophyly of Limnotyphloplanida, the clade roughly comprising Dalyelliidae, most Typhloplanidae and Temnocephalida, is well-established. This freshwater clade was already suggested by Jondelius and Thollesson [2] and Zamparo et al. [35] based on morphological data. Willems et al. [1] also retrieved these three freshwater clades within Neotyphloplanida, but could not corroborate limnotyphloplanid monophyly with 18S sequence data. The present study, however, strongly suggests a major ecological shift and a spectacular evolutionary radiation when the common ancestor of Temnocephalida, Dalyelliidae and Typhloplanidae invaded the limnic environment. This is supported by the fact that more than half of the currently known species occur in this environment and the majority of them belong to one of the limnotyphloplanid genera present in this study.

Dalyelliidae, Typhloplanidae and Temnocephalidae include some of the most species-rich rhabdocoel genera (e.g. *Gieysztoria*, *Castrada*, *Temnocephala*) possibly suggesting evolutionary radiation took place once freshwater environments were colonised. Many of these freshwater genera have representatives on all continents and some species seem to be widespread or even cosmopolitan (e.g. *Mesostoma lingua*, *Rhynchomesostoma rostratum*, *Gieysztoria cuspidata*). No less than 53 dalytyphloplanid species, mostly Protoplanellinae (Typhloplanidae), are limnoterrestrial [36]. This is remarkable, since the limnoterrestrial taxa *Acrochordonoposthia*, *Carcharodopharynx*, *Bryoplana*, *Protoplanella*, and *Krumbachia* form a polyphyletic assemblage suggesting multiple independent colonisations of limnoterrestrial habitats.

#### 1.2. From marine and brackish water to freshwater and back?

Surprisingly, our trees suggest several secondary returns to brackish water and marine habitats within Limnotyphloplanida. Species of *Halammovortex* and *Jensenia* are euryhaline. They appear as possible sister taxa of the limnic temnocephalids, but since many other dalyelliid genera that might be more closely-related to either Temnocephalida (e.g. *Varsoviella* and *Alexlutheria*; see further section 2.1) or *Halammovortex* and *Jensenia* have not been included in the analysis, the origins of symbiosis and a secondary return to brackish and marine environments remain highly speculative in this clade. In addition, four species of *Gieysztoria* exclusively occur in brackish water or marine environments (*G. expeditoides*, *G. maritima*, *G. reggae* and *G. subsalsa*; see [37]–[39]). These species were not available for this study, but their morphology suggests that they undoubtedly belong to *Gieysztoria*. Other dalyelliid species are limnic, but occur facultatively in brackish water (e.g. *M. armigera*, *M. fusca*, *G. cuspidata*, *G. knipovici*, *G. triquetra*; [39]). Similar examples of limnic species that may also inhabit brackish water of up to 5‰, can be found within the limnic Typhloplanidae (e.g. *Castrada hofmanni*, *C. intermedia*, *C. lanceola*, *Mesostoma lingua*, *Strongylostoma elongatum* and *Typhloplana viridata*) [39], [40]. This suggests that recolonisation of brackish habitats may be relatively easy and may have occurred independently in different clades.

#### 1.3. Freshwater invasions by thalassotyphloplanids and neodalyellids

In addition to the single-origin freshwater colonisation of the Limnotyphloplanida, independent shifts from marine to limnic environments also occurred within Thalassotyphloplanida and Neodalyellida. Many, predominantly marine genera in these clades have obligate or facultative brackish water representatives (Thalassotyphloplanida: *Beklemischeviella*, *Brinkmanniella*, *Byrsophlebs*, *Coronhelmis*, *Maehrenthalia*, *Promesostoma*, *Proxenetes*, *Ptychopera*, *Thalassoplanella*, *Trigonostomum*; Neodalyellida: *Baicalellia*, *Balgetia*, *Bresslauilla*, *Canetellia*, *Pogaina*, *Provortex*, *Pseudograffilla*, *Vejdovskya*; [39], [40]), but only few have become limnic (see [41] for an overview). The sole hollimnic thalassotyphloplanid is *Styloplanella strongylostomoides*, a species apparently confined to alpino-boreal bogs. Although only 18S data were available for this taxon, Bayesian analyses support it as a member of the Byrsophlebidae+(marine) Typhloplanidae clade (PP 1.0). Findenegg [42] already stressed its problematic position within Typhloplaninae and even expressed doubts on its position within Typhloplanidae. The presence of a stylet and the species' overall morphology is similar to that of other marine Typhloplanidae, suggesting a phylogenetic reassessment of the other marine representatives of this taxon is needed.

Obligate limnic neodalyellids are very rare. We included an unidentified species of Provorticidae (*Provorticidae* sp.) from Hawaiian freshwater ponds, with a morphology that was somewhat reminiscent of the poorly-described *Provortex sphagnorum*. Some marine and euryhaline neodalyellid species have also been found in limnic habitats. For *Bresslauilla relicta* many inland records from freshwater habitats are known [39], [43], while the euryhaline *Vejdovskya ignava* and *Coronhelmis multispinosus* locally invade the limnic zones of the Elbe estuary [39], [44], [45]. Several hypotheses on how these colonisations may have taken place, were tentatively suggested by Kolasa [41]. Euryhaline habitats such as estuaries, old land-locked seawater basins and salt marches could have acted as stepping stones between marine and limnic habitats. Possibly *Bresslauilla relicta* and the limnic provorticid from Hawaii, which are both closely related to many neokirgelline taxa that are euryhaline (*Baicalellia brevituba*, *Balgetia semicirculifera*, *Canetellia beauchampi*) or exclusively known from salt marshes (*Eldenia reducta*), fit into this hypothesis.

### 2. Symbiosis

Symbiotic taxa are scattered throughout several clades in our trees, confirming previous findings that symbiotic life strategies have evolved multiple times independently in both marine and freshwater dalytyphloplanids [1], [2]. Ecological and evolutionary triggers for the origins of symbiotic relations and the transition from facultative commensalism to obligate parasitism are far from clear in non-neodermatan flatworms in general and dalytyphloplanids in particular (for an interesting hypothesis see [46]).

#### 2.1. Temnocephalida

Temnocephalida, ectosymbionts of various freshwater invertebrates and chelonians, display nearly all intermediate steps of transition from commensalism to parasitism. As corroborated by our phylogenies, temnocephalids are in fact ectosymbiotic dalyelliids.

The phylogenetic position of Temnocephalida has always been controversial. Various studies linking them to Neodermata have later been rejected (see [47] and references therein). A close relationship between Dalyelliidae and Temnocephalida was suggested by Karling [48], who proposed the stylet morphology of *Alexlutheria* as an ancestral “template” for the Temnocephalida in general and for *Didymorchis* in particular. Later, a sister group relation between free-living Dalyelliidae and Temnocephalida was tentatively confirmed by Ehlers [49], Jondelius and Thollesson [2] and Zamparo et al. [35]. Ehlers and Sopott-Ehlers [50] also supported this point of view based on stylet morphology and the ultrastructural synapomorphies of *Jensenia* and the temnocephalids (duo-gland adhesive system and lamellated rhabdites). Although very different life strategies have been adopted by the free-living Dalyelliidae and by the ectosymbiotic Temnocephalida, the position of Temnocephalida within Dalyelliidae is plausible considering the very similar internal morphology of both taxa. As such, the temnocephalid genus *Didymorchis* has even been linked to either Dalyelliidae or Temnocephalida, but cladistical analyses of morphological data and ultrastructural studies on the epidermis confirms that it belongs to Temnocephalida [51], [52]. This temnocephalid relationship is fully supported by our DNA data. Several morphological synapomorphies have been defined for Temnocephalida, including a multisyncytial epidermis, a posterior adhesive organ, genito-intestinal communication and a split shaft of sperm cells [51], [53].

#### 2.2. Pterastericolidae

Pterastericolidae are parasites of starfish, which have been linked to different dalyellioid groups and even to Neodermata (see [2] and references therein, [54]–[56]). The latter hypothesis was, however, rejected by DNA sequence data [57] and different ultrastructural characters (spermiogenesis, sperm, protonephridia; see [58] and references therein). One pterastericolid species was included in the analyses of Willems et al. [1], which showed it to be firmly embedded within Neodalyellida as the sister taxon of the endosymbiotic graffillid *Graffilla buccinicola*. Our data confirm its position within Neodalyellida and also suggest that pterastericolids are closely related to some neokirgelline provorticids (*Canetellia*, *Baicalellia*) and some graffillids (*Graffilla*, *Pseudograffilla*).

#### 2.3. Graffillidae

This family is a polyphyletic amalgam of endosymbiotic and free-living species. *Graffilla buccinicola* lives in the digestive glands of carnivorous gastropods. It predominantly feeds on its host's partly-digested food and almost entirely depends on its host's digestive enzymes [59]. *Pseudograffilla arenicola* and a yet unidentified marine dalyellioid species (*Dalyellioida* “houdini” sp.), both free-living, emerge as the sister taxa of *G. buccinicola* in our topologies. *Pseudograffilla* is known to be microphagous rather than predatory, while for *Dalyellioida* “houdini” sp. stylets of other rhabdocoels were among its gut contents (Willems, unpublished observations). The fact that *Dalyellioida* “houdini” sp. is related to *G. buccinicola* and *P. arenicola* opens interesting perspectives for future research on the evolution of nutritional strategies in this group [60].

#### 2.4. Umagillidae

Umagillidae is the most species-rich taxon of symbiotic non-neodermatan flatworms [61], [62]. Umagillids are common endosymbionts of echinoderms, with few species also living inside sipunculids. They demonstrate a wide variety of feeding behaviours, ranging from endozoic predation of symbiotic protozoans to full endoparasitic feeding on its host's tissues. Little in-depth information (e.g. physiology, host specificity, nutritional preferences) is available about life history strategies of umagillids. Most species, including species of *Seritia* and *Wahlia* are intestinal, while species of *Anoplodium* are confined to the coelomic regions of its host. Cannon [61] suggests that species of *Anoplodium* must have been derived from intestinal inhabiting forms. With only three umagillid taxa represented in this study, none of these hypotheses could be corroborated. Yet, the phylogenetic position of Umagillidae confirms that symbiotic relations in marine rhabdocoels originated multiple times independently involving different nutritional strategies that eventually led to full endoparasitism in some clades.

### 3. Taxonomic implications

Our phylogenetic inferences have important implications for the taxonomy of rhabdocoels in general and of Dalytyphloplanida in particular. The addition of new taxa and a second gene fragment (28S rDNA) in this study compared to that of Willems et al. [1], largely confirms the new clades by Willems et al. [1], i.e. Neotyphloplanida, Neodalyellida and Thalassotyphloplanida. The monophyly of taxa that have long been regarded as such based on morphology (Dalyelliidae, Umagillidae, Pterastericolidae, Temnocephalida, Kytorhynchidae, Solenopharyngidae), is firmly supported by our DNA sequence data, while many of the uncertain taxa (Graffillidae, Provorticidae, Promesostomidae, Trigonostomidae, Typhloplanidae, Byrsophlebidae; see [2] for a morphological overview) are clearly not monophyletic in our trees. The division of Dalytyphloplanida in Neodalyellida and Neotyphloplanida is well-supported.

#### 3.1. Neodalyellida

The *Einarella+Trisaccopharynx*, *Anoplodium+Provortex* and *Graffilla+Pterastericola* clades suggested by Willems et al. [1], are confirmed and extended in the present analysis. The position of the promesostomid *Einarella argillophyla* as the sister group of Solenopharyngidae is one of the several indications of the polyphyly of Promesostomidae, of which other representatives are scattered throughout Thalassotyphloplanida (see below). Solenopharyngidae has been well diagnosed by Ehlers [63] and is likely monophyletic. However, our analysis included species of only one of the three recognised subfamilies (Solenopharynginae), except for *Solenopharyngidae* sp., which could not be placed in any subfamily because of a lack of data.

Provorticidae and Graffillidae are clearly not monophyletic, since species belonging to these families are scattered over the two other major neodalyellid clades. As some authors pointed out, the monophyly of Provorticidae is not supported by morphological synapomorphies [64], [65]. Moreover, the taxonomic mixing of Provorticidae and Graffillidae is not surprising, since both taxa were originally regarded as subfamilies of a single family that was first named Graffillidae, and later on Provorticidae [66]–[68]. The present taxonomic separation into Graffillidae and Provorticidae is solely based on the position of the gonopore [68], [69], a character considered of poor phylogenetic value by Karling [48], [70]. Two peculiar sister taxa of the “mixed” clade (see results), *Neodalyellida* sp. 1 and *Neodalyellida* sp. 2 could not be placed in any of the existing families. Their overall morphology suggests that they belong to one of the marine dalyellioid clades, but the presence of an anterior male copulatory organ with a stylet is a unique feature within dalytyphloplanids. Only graffillids also have a male copulatory organ in the anterior body half, though without a stylet. The provorticid *Eldenia reducta* is closely related to the free-living graffillid *Bresslauilla relicta*. However, *Eldenia reducta* has a caudal stylet accompanied by an accessory stylet, a feature that is unique within Provorticidae. The other well-delined taxa within the “mixed” clade, *Baicalellia*, *Balgetia*, *Canetellia* and *Pogaina*, are phylogenetically closely related. Traditionally they are grouped within Neokirgellinae, which is clearly not monophyletic because of the position of the Pterastericolidae.

Provorticinae (*Provortex*, *Vejdovskya*) is well-separated from the Neokirgellinae in our trees. This separation is also supported by morphology based on the relative position of prostate and seminal vesicle [70], [71]. The close relation between *Provortex* and *Vejdovskya*, the only difference being separate ovaries and vitellaria in *Provortex* as opposed to ovovitellaria in *Vejdovskya*, was already suggested by Luther [72] and Marcus [68] and is confirmed in our 18S and combined trees (no 28S sequences of *Provortex* available), though in a polytomy with the endosymbiotic Umagillidae in the concatenated analysis. Only one umagillid species (*Anoplodium stichopi*) was included in the study of Willems et al. [1], where it appeared as the sister taxon of *Provortex*. The close relationship of umagillids with Provorticinae was already discussed in Karling [48] and is now firmly supported by our molecular phylogenies.

#### 3.2. Thalassotyphloplanida

The 28S and combined data suggest that Kytorhynchidae is the sister taxon of all other thalassotyphloplanids. Kytorhynchidae have a glandular “false” proboscis derived from a permanent anterior terminal invagination of the body wall. The differentiation of the anterior tip into a proboscis-like structure is a feature they share with some other “typhloplanoids” (e.g. *Trigonostomum*, *Astrotorhynchus*, *Microvahine*, *Rhynchomesostoma*, *Adenorhynchus*, *Haplorhynchella*, *Microcalyptorhynchus*, *Prorhynchella*, *Pararhynchella*; [73]–[75]). The presence of a proper muscular proboscis is typical of Kalyptorhynchia. Based on “proboscis” structure and other morphological data, Rieger [75] placed Kytorhynchidae within the non-kalyptorhynch rhabdocoels. The present phylogenetic study confirms that kytorhynchids are indeed not kalyptorhynchs, but dalytyphloplanids.

The internal relationships of Thalassotyphloplanida show that many of the traditional marine typhloplanoid genera (*Trigonostomum*, *Promesostoma*, *Proxenetes*, *Ceratopera*) and subfamilies (Promesostominae, Paramesostominae, Trigonostominae) are monophyletic, but that the two largest traditional families are not. Indeed, both Trigonostomidae and Promesostomidae appear to be polyphyletic. In the cladogram, Trigonostomidae is represented by two clades: Trigonostominae, sistergroup to a clade consisting of the promesostomid taxa *Litucivis* (Adenorhynchinae) and *Cilionema* and *Coronhelmis* (Brinkmanniellinae), and Paramesostominae, which forms a well-supported sistergroup relationship with *Promesostoma* (Promesostominae, Promesostomidae). This Paramesostominae+*Promesostoma* clade is part of a larger unresolved clade containing among others *Brinkmanniella palmata* (Brinkmanniellinae, Promesostomidae), clearly showing the polyphyletic nature of the taxon Promesostomidae, a member of which is even found within Neodalyellida (*Einarella argillophyla*, see above). Another clade within the polytomy is formed by species of Byrsophlebidae, a number of marine Typhloplanidae and one freshwater typhloplanid, *Styloplanella strongylostomoides*. Both Typhloplanidae and Byrsophlebidae share the presence of one single ovary, but species of Byrsophlebidae have separate male and female genital pores, whereas species of Typhloplanidae have a common genital pore. Species of Typhloplanidae are nearly all limnic, but some, including *Thalassoplanella* and *Kaitalugia*, occur in brackish water and marine environments. Although Luther [76] classified *Thalassoplanella* into Typhloplanidae, he firmly expressed his reluctance to do so because of certain morphological features (e.g. the presence of a reduced second ovary). A similar situation applies to *Kaitalugia*, which Willems et al. [77] considered to be very closely related to *Thalassoplanella*. In addition, many authors [75], [78]–[81] state that the taxonomic position of other marine typhloplanid genera is doubtful (e.g. *Haloplanella*, *Gullmariella*, *Notomonoophorum*, *Thalassoplanina*, *Tauridella*, *Lioniella*, *Ruanis*, *Stygoplanellina*, *Pratoplana*, *Magnetia*, and *Haplodidymos*). With the presence of a stylet in the male genital system (unusual for Typhloplanidae) most of these brackish water and marine taxa appear similar to Byrsophlebidae, except that Byrsophlebidae have separate male and female genital pores.

This clearly shows that the higher-level phylogeny (i.e. above genus) of the taxa in question is highly in need of revision, which was already discussed by Karling et al. [73] and many of the above-mentioned authors (e.g. [75]).

#### 3.3. Limnotyphloplanida

The limnic dalyelliid genera *Castrella*, *Dalyellia*, *Microdalyellia*, and *Gieysztoria* are confirmed as clades. Only the Australian *G.* cf. *billabongensis* is positioned outside *Gieysztoria*. This species forms a separate clade with *Dalyelliidae* n. gen. n. sp., a yet unidentified species from India. Irrespective of whether or not this Indian taxon is the same as the Australian one, it is clear that based on our molecular data, both taxa are not part of the *Gieysztoria* clade. In general, intergeneric dalyelliid relations are far from clear, illustrated by the conflicting or unresolved position of several genera (e.g. *Castrella*, *Dalyellia*, *Microdalyellia*, *Pseudodalyellia*, *Gieysztoria*) in the 18S and 28S trees.

## Conclusions

Our phylogenies clearly show that: (a) dalytyphloplanids have their origins in the marine environment, (b) colonisation of a wide range of limnic and limnoterrestrial habitats took place when the common ancestor of all species of Limnotyphloplanida (comprising all Dalyelliidae, Temnocephalida, and nearly all limnic Typhloplanidae) escaped its marine environment; (c) this colonisation was followed by a spectacular radiation, resulting in speciose genera of Limnotyphloplanida, (d) some thalassotyphloplanids and neodalyellids also invaded limnic environments, though very sporadically and not followed by speciation events as in Limnotyphloplanida; (e) secondary returns to brackish water and marine environments occurred relatively frequently in several dalyelliid and typhloplanid taxa; (f) some well-diagnosed rhabdocoel families or subfamilies are monophyletic (freshwater Dalyelliidae, Umagillidae, Pterastericolidae, Temnocephalida, Solenopharyngidae, Trigonostominae, Paramesostominae, Promesostominae), while others are clearly polyphyletic (Graffillidae, Provorticidae, Promesostomidae, Trigonostomidae, Typhloplanidae, Byrsophlebidae); (g) Kytorhynchidae, an enigmatic “typhloplanoid” taxon, is most likely the sister taxon to all other thalassotyphloplanids; (h) *Carcharodopharynx* can be assigned to Typhloplanidae.

Although many new dalytyphloplanid clades can be formally recognised based on this molecular phylogeny, we provisionally refrain from erecting a new formal (DNA-based) classification. We believe that this should ideally be backed up by thorough morphological studies in which clear (syn)apomorphies for each newly-established clade are delineated.

## Supporting Information

Figure S1 **Majority-rule consensus tree from the Bayesian analysis of the 18S rDNA dataset.** Legend identical to Fig. 2.
(TIF)

Figure S2 **Majority-rule consensus tree from the Bayesian analysis of the 28S rDNA dataset.** Legend identical to Fig. 2.
(TIF)

Table S1 **List of species, sampling locations and amplification primers as used in this study.** Additional sequences that were taken from GenBank are also listed with their GenBank accession number and when known, their sampling location. M: marine; F: freshwater; B: brackish water; L: limnoterrestrial; S: symbiotic.
(DOC)

Table S2 **Identical sequences removed from the analyses.**
(DOC)

Table S3 **Test of substitution saturation with DAMBE v5.2.57 according to Xia's method for more than 32 OTUs.** Analyses performed on all sites with gaps treated as unknown data. Subsets of 4, 8, 16 and 32 OTUs were randomly sampled 60 times and the test was performed for each subset. Iss: simple index of substitution saturation; Iss,cSym: critical Iss assuming a symmetrical topology; Iss,cAsym: critical Iss assuming an asymmetrical topology. If Iss is significantly smaller than Iss,c, little substitution saturation is present. Although Iss(18S+28S) does not significantly differ from Iss,cAsym(18S+28S), asymetrical trees are highly unlikely for our datasets.
(DOC)
