# Supplementary figures and images for: A Comprehensive Molecular Phylogeny of Dalytyphloplanida (Platyhelminthes: Rhabdocoela) Reveals Multiple Escapes from the Marine Environment and Origins of Symbiotic Relationships
Source: PLoS One. 2013 Mar 25;8(3):e59917. doi: 10.1371/journal.pone.0059917 (PMC3607561; doi:10.1371/journal.pone.0059917)

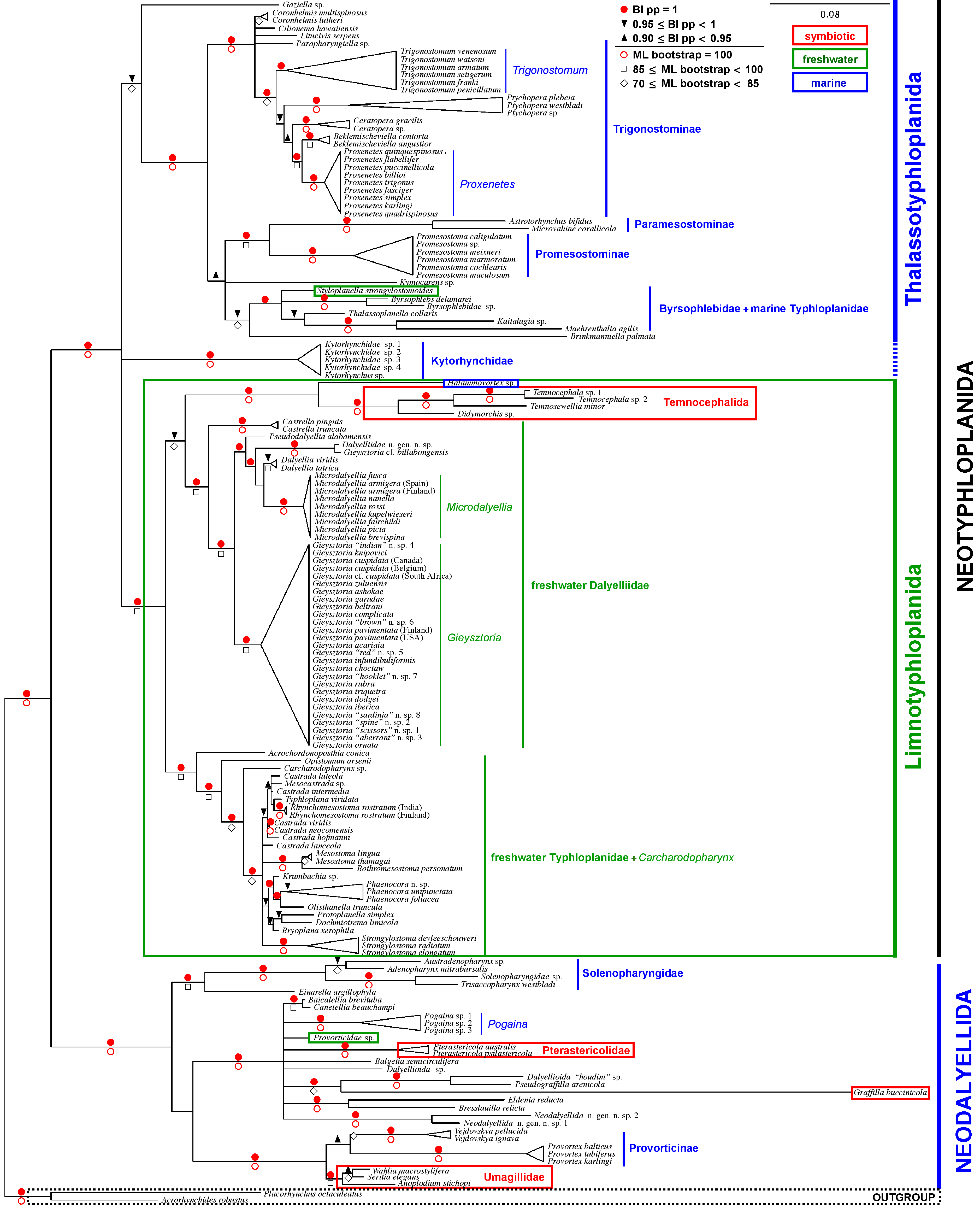

Supplement: Figure S1 — Majority-rule consensus tree from the Bayesian analysis of the 18S rDNA dataset. Legend identical to Fig. 2. (TIF) [file pone.0059917.s001.tif]

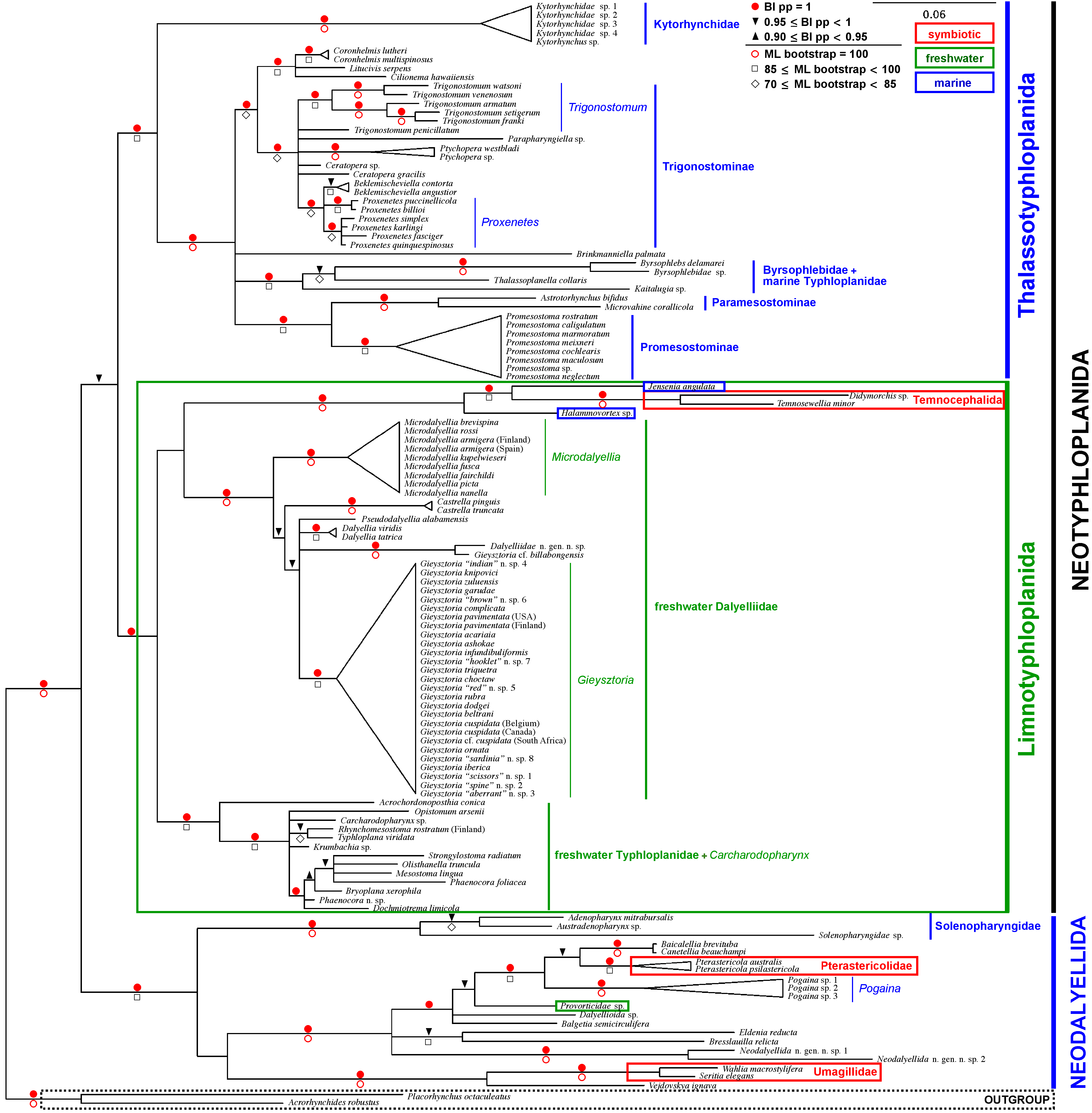

Supplement: Figure S2 — Majority-rule consensus tree from the Bayesian analysis of the 28S rDNA dataset. Legend identical to Fig. 2. (TIF) [file pone.0059917.s002.tif]
