# Supplementary material for: A Comprehensive Molecular Phylogeny of Dalytyphloplanida (Platyhelminthes: Rhabdocoela) Reveals Multiple Escapes from the Marine Environment and Origins of Symbiotic Relationships
Source: PLoS One. 2013 Mar 25;8(3):e59917. doi: 10.1371/journal.pone.0059917 (PMC3607561; doi:10.1371/journal.pone.0059917)
Supplement: Table S1 — List of species, sampling locations and amplification primers as used in this study. Additional sequences that were taken from GenBank are also listed with their GenBank accession number and when known, their sampling location. M: marine; F: freshwater; B: brackish water; L: limnoterrestrial; S: symbiotic. (DOC) [file pone.0059917.s003.doc]

| **Table S1.** List of species, sampling locations and amplification primers as used in this study. | | | | | |
| --- | --- | --- | --- | --- | --- |
| **Taxon** |  | **Sampling location** | **Coordinates** | **Primer pair \| Acc. number** | |
|  |  |  |  | **18S** | **28S** |
| **Kytorhynchidae Rieger, 1974** |  |  |  |  |  |
| *Kytorhynchus* sp. | M | Waimanalo beach, Oahu, Hawai’i, USA | 21°19’36”N; 157°40’59”W | TimA/TimB \| KC529400 | LSU5/LSUD6.3B \| KC52926 |
| *Kytorhynchidae* sp. 1 | M | Punta Negra, Sardinia, Italy | 40°57’12”N; 08°13’43”E | TimA/TimB \| KC529401 | LSU5/LSUD6.3B \| KC52927 |
| *Kytorhynchidae* sp. 2 | M | Alghero, Sardinia, Italy | 40°32’39”N; 08°19’13”E | TimA/TimB \| KC529402 | LSU5/LSUD6.3B \| KC52928 |
| *Kytorhynchidae* sp. 3 | M | Rota, Andalusia, Spain | 36°38’09”N; 06°23’50”W | TimA/TimB \| KC529403 | LSU5/LSUD6.3B \| KC52929 |
| *Kytorhynchidae* sp. 4 | M | Punta Negra, Sardinia, Italy | 40°57’12”N; 08°13’43”E | TimA/TimB \| KC529404 | LSU5/LSUD6.3B \| KC52930 |
| **Trigonostomidae Graff, 1905** |  |  |  |  |  |
| Trigonostominae Luther, 1948 |  |  |  |  |  |
| *Parapharyngiella* sp. | M | Doha, Qatar | 25°19’09”N; 51°32’16”E | TimA/TimB \| KC529405 | LSU5/LSUD6.3 \| KC529531 |
| *Proxenetes quinquespinosus* Ax, 1971 | M | Sylt, Germany | 55°02’06”N; 08°24’29”E | TimA/TimB \| KC529406 | LSU5/LSUD6.3 \| KC529532 |
| *Proxenetes billioi* Den Hartog, 1966 | B | Zwin, the Netherlands | 51°21’56”N; 03°22’18”E | TimA/TimB \| KC529407 | LSU5/LSUD6.3 \| KC529533 |
| *Proxenetes fasciger* Ehlers, 1974 | M | Sylt, Germany | 55°01’21”N; 08°26’25”E | Prom18SFb/Prom18SRb (DK18S65) \| KC529408 | LSU5/LSUD6.3 \| KC529534 |
| *Proxenetes trigonus* Ax, 1960 | M | Sylt, Germany | 55°01’21”N; 08°26’25”E | AY775768 | NA |
| *Proxenetes karlingi* Luther, 1943 | B | Zwin, the Netherlands | 51°21’56”N; 03°22’18”E | TimA/TimB \| KC529409 | LSU5/LSUD6.3 \| KC529535 |
| *Proxenetes quadrispinosus* Den Hartog, 1966 | M | Sylt, Germany | 55°01’21”N; 08°26’25”E | AY775766 | NA |
| *Proxenetes flabellifer* Jensen, 1878 | M | Oostende, Belgium | NA | AY775764 | NA |
| *Proxenetes simplex* Luther, 1948 | B | Zwin, the Netherlands | 51°21’56”N; 03°22’18”E | TimA/TimB \| KC529410 | LSU5/LSUD6.3 \| KC529536 |
| *Proxenetes puccinellicola* Ax, 1960 | B | Zwin, the Netherlands | 51°21’56”N; 03°22’18”E | TimA/TimB \| KC529411 | LSU5/LSUD6.3 \| KC529537 |
| *Beklemischeviella angustior* Luther, 1943 | B | Hanko, Finland | 59°49’50”N; 23°08’33”E | TimA/TimB \| KC529412 | LSU5/LSUD6.3 \| KC529538 |
| *Beklemischeviella contorta* (Beklemischew, 1927) Luther, 1943 | B | Hanko, Finland | 59°49’21”N; 22°58’21”E | TimA/TimB \| KC529413 | LSU5/LSUD6.3 \| KC529539 |
| *Trigonostomum penicillatum* (Schmidt, 1857) Micoletzky, 1910 | M | Porto Torres, Sardinia, Italy | 40°49’33”N; 08°26’36”E | Prom18Fb/Prom18Rb (DK18S65) \| KC529414 | LSU5/LSUD6.3 \| KC529540 |
| *Trigonostomum watsoni* Willems et al., 2004 | M | iSimangaliso, KwaZulu-Natal, South Africa | 28°16’50”S; 32°29’06”E | TimA/TimB \| KC529415 | LSU5/LSUD6.3B \| KC529541 |
| *Trigonostomum franki* Willems et al., 2004 | M | Aguada Fort, Goa, India | 15°29’38”N; 73°46’03”E | Prom18Fb/Prom18Rb (DK18S65) \| KC529416 | LSU5/LSUD6.3 \| KC529542 |
| *Trigonostomum venenosum* (Uljanin, 1870), Meixner, 1924 | M | Punta Negra, Sardinia, Italy | 40°57’12”N; 08°13’43”E | TimA/TimB \| KC529417 | LSU5/LSUD6.3 \| KC529543 |
| *Trigonostomum setigerum* Schmidt, 1852 | M | Punta Negra, Sardinia, Italy | 40°57’12”N; 08°13’43”E | Prom18Fb/Prom18Rb (DK18S65) \| KC529418 | LSU5/LSUD6.3 \| KC529544 |
| *Trigonostomum armatum* (Jensen, 1878) Gamble, 1900 | M | Inre Vattenholmen, Bohuslän, Sweden | 58°52’36”N; 11°06’40”E – 58°52’35”N; 11°06’46”E | TimA/TimB, S30/5FK, 4FB/1806R \| KC529419 | LSU5/LSUD6.3 \| KC529545 |
| *Ptychopera westbladi* (Luther, 1943) Den Hartog, 1964 | B | Zwin, Belgium | NA | AY775770 | NA |
| *Ptychopera westbladi* (Luther, 1943) Den Hartog, 1964 | B | Colijnsplaat, Zealand, the Netherlands | 51°35’38”N; 03°52’17”E | NA | LSU5/LSUD6.3 \| KC529546 |
| *Ptychopera plebeia* (Beklemischew, 1927) Den Hartog, 1964 | M | Agia Trias, Thessaloniki, Greece | NA | AY775769 | NA |
| *Ptychopera* sp. | B | Pouhala marsh, Oahu, Hawai’i, USA | 21°22’36”N; 158°00’17”W | TimA/TimB \| KC529420 | LSU5/LSUD6.3 \| KC529547 |
| *Ceratopera* sp. | M | Anjuna beach, Goa, India | 15°34’03”N; 73°44’29”E | TimA/TimB \| KC529421 | LSU5/LSUD6.3 \| KC529548 |
| *Ceratopera gracilis* (Graff, 1882) Den Hartog, 1964 | M | Alghero, Sardinia, Italy | 40°32’39”N; 08°19’13”E | TimA/TimB \| KC529422 | LSU5/LSUD6.3 \| KC529549 |
| Paramesostominae Luther, 1948 |  |  |  |  |  |
| *Microvahine corallicola* Karling et al., 1972 | M | East Point, Northern Territory, Australia | 12°24’16”S; 130°48’49”E | Prom18Fb/Prom18Rb (DK18S65) \| KC529423 | LSU5/LSUD6.3B \| KC529550 |
| *Astrotorhynchus bifidus* (McIntosh, 1874) Graff, 1905 | M | NA | NA | AJ312270 | NA |
| *Astrotorhynchus bifidus* (McIntosh, 1874) Graff, 1905 | M | Gåsevik, Sweden | 58°14’46”N; 11°26’13”E | NA | LSU5/LSUD6.3B \| KC529551 |
| **Promesostomidae Den Hartog, 1964** |  |  |  |  |  |
| *Gaziella* sp. | M | Perea, Thessaloniki, Greece | NA | AY775776 | NA |
| Adenorhynchinae Ax and Heller, 1970 |  |  |  |  |  |
| *Litucivis serpens* Ax and Heller, 1970 | M | Sylt, Germany | 55°00’55”N; 08°26’19”E | AY775758 | LSU5/LSUD6.3 \| KC529552 |
| Brinkmanniellinae Luther, 1948 |  |  |  |  |  |
| *Brinkmanniella palmata* Karling, 1986 | M | Alghero, Sardinia, Italy | 40°32’39”N; 08°19’13”E | TimA/TimB \| KC529424 | LSU5/LSUD6.3B \| KC529553 |
| *Kymocarens* sp. | M | Arambol, Goa, India | 15°42’10”N; 73°41’40”E | TimA/TimB (DK18S35cycli) \| KC529425 | NA |
| *Coronhelmis lutheri* Ax, 1951 | M | Hallands Väderö, Skåne, Sweden | 56°25’57”N; 12°34’18”E | – \| KC529426 | – \| KC529554 |
| *Coronhelmis multispinosus* Luther, 1948 | M | Hanko, Finland | 59°49’21”N; 22°58’21”E | TimA/TimB \| KC529427 | LSU5/LSUD6.3 \| KC529555 |
| *Cilionema hawaiiensis* Karling et al., 1972 | M | Waimanalo beach, Oahu, Hawai’i, USA | 21°19’36”N; 157°40’59”W | TimA/TimB \| KC529428 | LSU5/LSUD6.3 \| KC529556 |
| *Einarella argillophyla* Luther, 1948 | M | Gullmaren, Sweden | 58°17’21”N; 11°30’23”E | AY775757 | NA |
| Promesostominae Luther, 1948 |  |  |  |  |  |
| *Promesostoma cochlearis* Karling, 1935 | B | Långskär, Raseborg, Finland | 59°49’08”N; 23°15’27”E | Prom18Fb/Prom18Rb (DK18S65) \| KC529429 | LSU5/LSUD6.3 \| KC529557 |
| *Promesostoma marmoratum* (Schultze, 1851) Graff, 1882 | B | Hanko, Finland | 59°49’10”N; 23°05’02”E | Prom18Fb/Prom18Rb (DK18S65) \| KC529430 | LSU5/LSUD6.3 \| KC529558 |
| *Promesostoma* sp. | M | Kaneohe bay, Oahu, Hawai’i, USA | 21°30’47”N; 157°50’09”W | Prom18Fb/Prom18Rb (DK18S65) \| KC529431 | LSU5/LSUD6.3 \| KC529559 |
| *Promesostoma caligulatum* Ax, 1952 | M | Sylt, Germany | 55°02’06”N; 08°24’29”E | Prom18Fb/Prom18Rb (DK18S65) \| KC529432 | LSU5/LSUD6.3 \| KC529560 |
| *Promesostoma meixneri* Ax, 1951 | M | Sylt, Germany | 55°01’27”N; 08°26’13”E | Prom18Fb/Prom18Rb (DK18S65) \| KC529433 | LSU5/LSUD6.3 \| KC529561 |
| *Promesostoma maculosum* Ax, 1956 | M | Rota, Andalusia, Spain | 36°38’09”N; 06°23’50”W | Prom18Fb/Prom18Rb (DK18S65) \| KC529434 | LSU5/LSUD6.3 \| KC529562 |
| *Promesostoma rostratum* Ax, 1951 | M | Saltö, Bohuslän, Sweden | 58°52’08”N; 11°08’28”E | NA | LSU5/LSUD6.3 \| KC529563 |
| *Promesostoma neglectum* Karling, 1967 | M | Tjärnö, Bohuslän, Sweden | 58°52’51”N; 11°08’30”E | NA | LSU5/LSUD6.3 \| KC529564 |
| **Byrsophlebidae Graff, 1905** |  |  |  |  |  |
| *Maehrenthalia agilis* (Levinsen, 1879) Graff, 1905 | M | NA | NA | AJ312273 | NA |
| *Byrsophlebs delamarei* (Ax, 1956) Karling, 1985 | B | Pyrénées-Orientales, France | NA | TimA/TimB \| KC529435 | LSU5/LSUD6.3B \| KC529565 |
| *Byrsophlebidae* sp. | M | Torres, Rio Grande do Sul, Brazil | 29°21’06”S; 49°43’50”E | TimA/TimB \| KC529436 | LSU5/LSUD6.3B \| KC529566 |
| **Temnocephalidae Monticelli, 1899** |  |  |  |  |  |
| *Temnosewellia minor* (Haswell, 1888) Damborenea and Cannon, 2001 | FS | Armidale, Australia | NA | AY157183 | AY157164 |
| *Temnocephala* sp. 1 | FS | NA | NA | AJ012520 | NA |
| *Temnocephala* sp. 2 | FS | NA | NA | AF051332 | NA |
| *Didymorchis* sp. | FS | Queensland, Australia | NA | AY157182 | AY157163 |
| **Dalyelliidae Graff, 1908** |  |  |  |  |  |
| *Halammovortex* sp. | B | Pyrénées-Orientales, France | NA | TimA/TimB \| KC529437 | LSU5/LSUD6.3 \| KC529567 |
| *Jensenia angulata* (Jensen, 1878) Graff, 1882 | M | Colijnsplaat, Zealand, the Netherlands | 51°35’38”N; 03°52’17”E | NA | LSU5/LSUD6.3 \| KC529568 |
| *Castrella pinguis* (Silliman, 1884) Fuhrmann, 1900 | F | Pryor Branch, Alabama, USA | 34°39’31”N; 86°58’22”W | TimA/TimB \| KC529438 | LSU5/LSUD6.3 \| KC529569 |
| *Castrella truncata* (Abildgaard, 1789) Hofsten, 1907 | F | Dundas Valley, Ontario, Canada | 43°15’08”N; 79°58’44”W | TimA/TimB \| KC529439 | LSU5/LSUD6.3 \| KC529570 |
| *Pseudodalyellia alabamensis* Van Steenkiste et al., 2011 | F | Akron, Alabama, USA | 32°48’27”N; 87°48’15”W | TimA/TimB \| KC529440 | LSU5/LSUD6.3 \| KC529571 |
| *Dalyelliidae* n. gen. n. sp. | F | Mayem lake, Goa, India | 15°34’33”N; 73°56’32”E | TimA/TimB \| KC529441 | LSU5/LSUD6.3 \| KC529572 |
| *Gieysztoria* cf. *billabongensis* Jondelius, 1997 | F | Girraween, Northern Territory, Australia | 12°30’57”S; 131°04’48”E | TimA/TimB (DK18S35cycli) \| KC529442 | LSU5/LSUD6.3 (DK28S35cycli) \| KC529573 |
| *Dalyellia tatrica* Gieysztor, 1934 | F | Kraubath an der Mur, Styria, Austria | 47°20’39”N; 14°53’14”E | TimA/TimB \| KC529443 | LSU5/LSUD6.3 \| KC529574 |
| *Dalyellia viridis* (Shaw, 1791) Graff, 1904 | F | Diepenbeek, Limburg, Belgium | 50°55’41”N; 05°23’30”E | TimA/TimB \| KC529444 | LSU5/LSUD6.3 \| KC529575 |
| *Microdalyellia kupelwieseri* (Meixner, 1915) Ruebush and Hayes, 1939 | F | Torfbroek, Belgium | 50°55’34”N; 04°32’36”E | TimA/TimB \| KC529445 | LSU5/LSUD6.3 \| KC529576 |
| *Microdalyellia picta* (Schmidt, 1848) Gieysztor, 1938 | F | Fjällfotasjön, Skåne, Sweden | 55°31’39”N; 13°17’37”E | – \| KC529446 | LSU5/LSUD6.3 \| KC529577 |
| *Microdalyellia fairchildi* (Graff, 1911) Ruebush and Hayes, 1939 | F | Pojo, Finland | 60°05’30”N; 23°31’46”E | TimA/TimB \| KC529447 | LSU5/LSUD6.3 \| KC529578 |
| *Microdalyellia rossi* (Graff, 1911) Gieysztor, 1938 | F | Summit bog, Ontario, Canada | 43°13’40”N; 80°03’15”W | TimA/TimB \| KC529448 | LSU5/LSUD6.3 \| KC529579 |
| *Microdalyellia nanella* (Beklemischew, 1921) Gieysztor, 1939 | F | Yddingesjön, Skåne, Sweden | 55°33’15”N; 13°15’18”E | TimA/TimB, S30/5FK, 4FB/1806R \| KC529449 | LSU5/LSUD6.3 \| KC529580 |
| *Microdalyellia brevispina* (Hofsten, 1911) Gieysztor, 1939 | F | Handöl, Jämtland, Sweden | 63°15’34”N; 12°26’49”E | TimA/TimB, S30/5FK, 4FB/1806R \| KC529450 | LSU5/LSUD6.3 \| KC529581 |
| *Microdalyellia armigera* (Schmidt, 1861) Gieysztor, 1938 | F | Pojo, Finland | 60°05’30”N; 23°31’46”E | TimA/TimB \| KC529451 | LSU5/LSUD6.3 \| KC529582 |
| *Microdalyellia armigera* (Schmidt, 1861) Gieysztor, 1938 | F | Donaña NP, Andalusia, Spain | 36°58’50”N; 06°29’11”W | TimA/TimB \| KC529452 | LSU5/LSUD6.3 \| KC529583 |
| *Microdalyellia fusca* (Fuhrmann, 1894) Ruebush and Hayes, 1939 | F | Raseborg, Finland | 59°55’35”N; 23°25’44”E | TimA/TimB \| KC529453 | LSU5/LSUD6.3 \| KC529584 |
| *Gieysztoria* “scissors” n. sp. 1 | F | Middle Point, Northern Territory, Australia | 12°39’04”S; 131°19’23”E | TimA/TimB \| KC529454 | LSU5/LSUD6.3 \| KC529585 |
| *Gieysztoria* “spine” n. sp. 2 | F | iSimangaliso, KwaZulu-Natal, South Africa | 28°09’27”S; 32°31’35”E | TimA/TimB \| KC529455 | LSU5/LSUD6.3 \| KC529586 |
| *Gieysztoria* “aberrant” n. sp. 3 | F | Hawson, South Australia, Australia | 34°41’19”S; 135°47’56”E | TimA/TimB (DK18S35cycli) \| KC529456 | LSU5/LSUD6.3 \| KC529587 |
| *Gieysztoria* cf. *cuspidata* (Schmidt, 1861) Ruebush and Hayes, 1939 | F | iSimangaliso, KwaZulu-Natal, South Africa | 28°21’40”S; 32°20’54”E | TimA/TimB \| KC529457 | LSU5/LSUD6.3 \| KC529588 |
| *Gieysztoria cuspidata* (Schmidt, 1861) Ruebush and Hayes, 1939 | F | Lommel, Limburg, Belgium | 51°14’44”N; 05°17’22”E | TimA/TimB \| KC529458 | LSU5/LSUD6.3 \| KC529589 |
| *Gieysztoria cuspidata* (Schmidt, 1861) Ruebush and Hayes, 1939 | F | Cootes Paradise, Ontario, Canada | 43°16’08”N; 79°54’31”W | TimA/TimB \| KC529459 | LSU5/LSUD6.3 \| KC529590 |
| *Gieysztoria ornata* (Hofsten, 1907) Ruebush and Hayes, 1939 | F | Schwarzensee, Styria, Austria | 47°17’17”N; 13°52’05”E | TimA/TimB \| KC529460 | LSU5/LSUD6.3 \| KC529591 |
| *Gieysztoria iberica* Van Steenkiste et al., 2011 | FB | Donaña NP, Andalusia, Spain | 36°54’50”N; 6°17’48”W | TimA/TimB \| KC529461 | LSU5/LSUD6.3 \| KC529592 |
| *Gieysztoria* “sardinia” n. sp. 8 | F | Platamona, Sardinia, Italy | 40°48’59”N; 08°28’14”E | TimA/TimB \| KC529462 | LSU5/LSUD6.3 \| KC529593 |
| *Gieysztoria knipovici* (Beklemischew, 1953) Luther, 1955 | B | Velim, Goa, India | 15°09’37”N; 73°57’34”E | TimA/TimB \| KC529463 | LSU5/LSUD6.3 \| KC529594 |
| *Gieysztoria* “indian” n. sp. 4 | F | Monzi, KwaZulu-Natal, South Africa | 28°28’02”S; 32°15’47”E | TimA/TimB \| KC529464 | LSU5/LSUD6.3 \| KC529595 |
| *Gieysztoria zuluensis* Van Steenkiste et al., acc. | F | Monzi, KwaZulu-Natal, South Africa | 28°27’57”S; 32°17’36”E | TimA/TimB \| KC529465 | LSU5/LSUD6.3 \| KC529596 |
| *Gieysztoria ashokae* Van Steenkiste et al., acc. | F | Parcem, Goa, India | 15°39’49”N; 73°46’33”E | TimA/TimB (DK18S45) \| KC529466 | LSU5/LSUD6.3 (DK28S35cycli) \| KC529597 |
| *Gieysztoria garudae* Van Steenkiste et al., acc. | F | Corlim, Goa, India | 15°29’33”N; 73°55’50”E | TimA/TimB \| KC529467 | LSU5/LSUD6.3 \| KC529598 |
| *Gieysztoria infundibuliformis* (Fuhrmann, 1894) Ruebush and Hayes, 1939 | F | Spåime, Jämtland, Sweden | 63°2’48”N; 12°21’24”E | – \| KC529468 | – \| KC529599 |
| *Gieysztoria* “red” n. sp. 5 | F | Kawainui, Oahu, Hawai’i, USA | 21°23’33”N; 157°45’43”W | TimA/TimB \| KC529469 | LSU5/LSUD6.3 \| KC529600 |
| *Gieysztoria acariaia* Marcus, 1946 | F | Nova Santa Rita, Rio Grande do Sul, Brazil | 29°46’30”S; 51°18’26”W | TimA/TimB (DK18S35cycli) \| KC529470 | LSU5/LSUD6.3 \| KC529601 |
| *Gieysztoria pavimentata* (Beklemischew, 1926) Ruebush and Hayes, 1939 | F | Pojo, Finland | 60°05’30”N; 23°31’46”E | TimA/TimB \| KC529471 | LSU5/LSUD6.3 \| KC529602 |
| *Gieysztoria pavimentata* (Beklemischew, 1926) Ruebush and Hayes, 1939 | F | Blackwell Run, Alabama, USA | 34°33’51”N; 86°46’43”W | TimA/TimB \| KC529472 | LSU5/LSUD6.3 \| KC529603 |
| *Gieysztoria complicata* (Fuhrmann, 1914) Ruebush and Hayes, 1939 | F | São Sebastião do Caí, Rio Grande do Sul, Brazil | 29°36’22”S; 51°22’00”W | TimA/TimB (DK18S35cycli) \| KC529473 | LSU5/LSUD6.3 \| KC529604 |
| *Gieysztoria* “brown” n. sp. 6 | F | Tramandaí, Rio Grande do Sul, Brazil | 29°58’10”S; 50°13’50”W | TimA/TimB \| KC529474 | LSU5/LSUD6.3 \| KC529605 |
| *Gieysztoria beltrani* (Gieysztor, 1931) Ruebush and Hayes, 1939 | F | Donaña NP, Andalusia, Spain | 36°49’27”N; 06°21’40”W | TimA/TimB \| KC529475 | LSU5/LSUD6.3 \| KC529606 |
| *Gieysztoria choctaw* Van Steenkiste et al., 2011 | F | Haines Island, Alabama, USA | 31°43’26”N; 87°28’47”W | TimA/TimB \| KC529476 | LSU5/LSUD6.3 \| KC529607 |
| *Gieysztoria* “hooklet” n. sp. 7 | F | Tramandaí, Rio Grande do Sul, Brazil | 29°58’10”S; 50°13’51”W | TimA/TimB \| KC529477 | LSU5/LSUD6.3 \| KC529608 |
| *Gieysztoria triquetra* (Fuhrmann, 1894) Ruebush and Hayes, 1939 | F | Tramandaí, Rio Grande do Sul, Brazil | 29°58’10”S; 50°13’50”W | TimA/TimB \| KC529478 | LSU5/LSUD6.3 \| KC529609 |
| *Gieysztoria dodgei* (Graff, 1911) Ruebush and Hayes, 1939 | F | Richland, Michigan, USA | 42°24’57”N; 85°26’22”W | TimA/TimB (DK18S35cycli) \| KC529479 | LSU5/LSUD6.3 (DK28S35cycli) \| KC529610 |
| *Gieysztoria rubra* (Fuhrmann, 1894) Ruebush and Hayes, 1939 | F | Albrunna Lund, Öland, Sweden | 56°19’4”N; 16°24’42”E | – \| KC529480 | LSU5/LSUD6.3 \| KC529611 |
| **Carcharodopharyngidae Bresslau, 1933** |  |  |  |  |  |
| *Carcharodopharynx* sp. | L | Klöch, Styria, Austria | 46°45’04”N; 15°56’38”E | TimA/TimB \| KC529481 | LSU5/LSUD6.3 \| KC529612 |
| **Typhloplanidae Graff, 1905** |  |  |  |  |  |
| Typhloplaninae Bresslau, 1933 |  |  |  |  |  |
| *Kaitalugia* sp. | M | East Point, Northern Territory, Australia | 12°24’16”S; 130°48’49”E | Prom18Fb/Prom18Rb (DK18S65) \| KC529482 | LSU5/LSUD6.3B \| KC529613 |
| *Styloplanella strongylostomoides* Findenegg, 1924 | F | Diepenbeek, Belgium | NA | AY775771 | NA |
| *Thalassoplanella collaris* Luther, 1946 | B | Långskär, Raseborg, Finland | 59°49’08”N; 23°15’27”E | TimA/TimB \| KC529483 | LSU5/LSUD6.3B \| KC529614 |
| *Typhloplana viridata* (Abildgaard, 1789) Luther, 1904 | F | Lepinjärvi, Raseborg, Finland | 60°03’08”N; 23°40’19”E | TimA/TimB \| KC529484 | LSU5/LSUD6.3 \| KC529615 |
| *Strongylostoma elongatum* Hofsten, 1907 | F | Diepenbeek, Belgium | NA | AY775771 | NA |
| *Strongylostoma radiatum* Müller, 1774 | F | Tingstädeträsk, Gotland, Sweden | 57°44’17”N; 18°37’29”E | – \| KC529485 | – \| KC529616 |
| *Strongylostoma devleeschouweri* Van Steenkiste et al., 2011 | F | Donaña NP, Andalusia, Spain | 36°58’49”N; 06°28’56”W | TimA/TimB \| KC529486 | NA |
| Protoplanellinae Reisinger, 1924 |  |  |  |  |  |
| *Acrochordonoposthia conica* Reisinger, 1924 | L | Belsele, East Flanders, Belgium | 51°09’13”N; 04°04’19”E | TimA/TimB \| KC529487 | LSU5/LSUD6.3 \| KC529617 |
| *Krumbachia* sp. | L | Dundas Valley, Ontario, Canada | 43°14’20”N; 79°59’17”W | TimA/TimB \| KC529488 | LSU5/LSUD6.3 \| KC529618 |
| *Bryoplana xerophila* Van Steenkiste et al., 2010 | L | Florence, Alabama, USA | 34°48’33”N; 87°40’04”W | TimA/TimB \| KC529489 | LSU5/LSUD6.3 \| KC529619 |
| *Protoplanella simplex* Reisinger, 1924 | L | La Puebla del Rio, Andalusia, Spain | 37°13’39”N; 06°10’52”W | TimA/TimB \| KC529490 | NA |
| Opistominae Luther, 1963 |  |  |  |  |  |
| *Opistomum arsenii* Nasonov, 1917 | F | Väster Dalsvallen, Jämtland, Sweden | 63°13’7”N; 12°24’35”E | TimA/TimB, S30/5FK, 4FB/1806R \| KC529491 | LSU5/LSUD6.3 \| KC529620 |
| Phaenocorinae Wahl, 1910 |  |  |  |  |  |
| *Phaenocora foliacea* Böhmig, 1914 | F | Mayem lake, Goa, India | 15°34’33”N; 73°56’32”E | TimA/TimB \| KC529492 | LSU5/LSUD6.3 \| KC529621 |
| *Phaenocora unipunctata* (Ørsted, 1843) Bendl, 1908 | F | De Maten, Diepenbeek, Belgium | NA | AY775762 | NA |
| *Phaenocora* n. sp. | F | Cootes Paradise, Ontario, Canada | 43°16’03”N; 79°55’13”W | TimA/TimB \| KC529493 | LSU5/LSUD6.3 \| KC529622 |
| Olisthanellinae Bresslau, 1933 |  |  |  |  |  |
| *Olisthanella truncula* (Schmidt, 1858) Voigt, 1892 | F | Donaña NP, Andalusia, Spain | 36°58’50”N; 06°29’11”W | TimA/TimB \| KC529494 | LSU5/LSUD6.3 \| KC529623 |
| Ascophorinae Findenegg, 1924 |  |  |  |  |  |
| *Dochmiotrema limicola* Hofsten, 1907 | F | Stekene, East Flanders, Belgium | 51°14’32”N; 04°05’03”E | TimA/TimB \| KC529495 | LSU5/LSUD6.3 \| KC529624 |
| Rhynchomesostominae Bresslau, 1933 |  |  |  |  |  |
| *Castrada lanceola* Braun, 1885 | F | Abisko, Norrbotten, Sweden | NA | AY775751 | NA |
| *Castrada hofmanni* Braun, 1885 | F | Spåime, Jämtland, Sweden | 63°2’48”N; 12°21’24”E | TimA/TimB, S30/5FK, 4FB/1806R \| KC529496 | NA |
| *Castrada luteola* Hofsten, 1907 | F | Abisko, Norrbotten, Sweden | NA | AY775752 | NA |
| *Castrada intermedia* (Volz, 1898) Luther, 1904 | F | Tingstädeträsk, Gotland, Sweden | 57°44’17”N; 18°37’29”E | – \| KC529497 | NA |
| *Castrada neocomensis* Volz, 1898 | F | Väster Dalsvallen, Jämtland, Sweden | 63°13’07”N; 12°24’35”E | – \| KC529498 | NA |
| *Castrada viridis* Volz, 1898 | F | Abisko, Norrbotten, Sweden | NA | AY775753 | NA |
| *Mesocastrada* sp. Volz, 1898 | F | NA | NA | U70081 | NA |
| *Rhynchomesostoma rostratum* (Müller, 1774) Luther, 1904 | F | Långskär, Raseborg, Finland | 59°49’09”N; 23°15’36”E | TimA/TimB \| KC529499 | LSU5/LSUD6.3 \| KC529625 |
| *Rhynchomesostoma rostratum* (Müller, 1774) Luther, 1904 | F | Mayem lake, Goa, India | 15°34’33”N; 73°56’32”E | TimA/TimB \| KC529500 | NA |
| Mesostominae Ehrenberg, 1836 |  |  |  |  |  |
| *Mesostoma thamagai* Artois et al., 2004 | F | Thamaga, Kweneng, Botswana | 24°41’50”S; 25°31’00”E | AY775760 | NA |
| *Mesostoma lingua* (Abildgaard, 1789) Graff, 1882 | F | Abisko, Norrbotten, Sweden | NA | AY775759 | NA |
| *Mesostoma lingua* (Abildgaard, 1789) Graff, 1882 | F | Doñana NP, Andalucia, Spain | 36°57’28”N; 06°26’59”W | NA | LSU5/LSUD6.3 \| KC529626 |
| *Bothromesostoma personatum* (Schmidt, 1848) Braun, 1885 | F | – | – | TimA/TimB, S30/5FK, 4FB/1806R \| KC529501 | NA |
| **Provorticidae Beklemischew, 1927** |  |  |  |  |  |
| *Eldenia reducta* Ax, 2008 | B | Zwin, the Netherlands | 51°21’56”N; 03°22’18”E | TimA/TimB \| KC529502 | Neodal28SFa/Neodal28SRb (DK28S65) \| KC529627 |
| Neokirgellinae Oswald et al., 2010 |  |  |  |  |  |
| *Balgetia semicirculifera* Karling in Luther, 1962 | M | Sylt, Germany | 55°01’34”N; 08°25’52”E | TimA/TimB \| KC529503 | LSU5/LSUD6.3 \| KC529628 |
| *Canetellia beauchampi* Ax, 1956 | B | Tjäreskäret, Västerbotten, Sweden | 63°28’2”N; 19°46’3”E | – \| KC529504 | – \| KC529629 |
| *Baicalellia brevituba* (Luther, 1921) Nasonov, 1930 | B | Zwin, the Netherlands | 51°21’56”N; 03°22’18”E | Neodal18SF/Neodal18SR (DK18S65) \| KC529505 | Neodal28SFa/Neodal28SRb (DK28S65) \| KC529630 |
| *Pogaina* sp. 3 | M | Kaneohe bay, Oahu, Hawai’i, USA | 21°30’47”N; 157°50’09”W | Neodal18SF/Neodal18SR (DK18S65) \| KC529506 | Neodal28SFa/Neodal28SRb (DK28S65) \| KC529631 |
| *Pogaina* sp. 1 | M | iSimangaliso, KwaZulu-Natal, South Africa | 28°23’44”S; 32°25’28”E | TimA/TimB \| KC529507 | LSU5/LSUD6.3 \| KC529632 |
| *Pogaina* sp. 2 | M | Poonindie, South Australia, Australia | 34°35’31”S; 135°54’15”E | Neodal18SF/Neodal18SR (DK18S65) \| KC529508 | Neodal28SFa/Neodal28SRb (DK28S65) \| KC529633 |
| Provorticinae Luther, 1962 |  |  |  |  |  |
| *Provorticidae* sp. | F | Waimea valley, Oahu, Hawai’i, USA | 21°38’06”N; 158°03’14”W | Neodal18SF/Neodal18SR (DK18S65) \| KC529509 | LSU5/LSUD6.3 \| KC529634 |
| *Provortex karlingi* Ax, 1951 | B | Hallands Väderö, Skåne, Sweden | 56°26’00”N; 12°34’26”E | – \| KC529510 | NA |
| *Provortex tubiferus* Luther, 1948 | M | Bohuslän, Sweden | NA | AJ312269 | NA |
| *Provortex balticus* (Schultze, 1851) Graff, 1882 | B | Sandhammaren, Skåne, Sweden | 55°23’07”N; 14°11’57”E | – \| KC529511 | NA |
| *Vejdovskya pellucida* (Schultze, 1851) Graff, 1913 | B | Hallands Väderö, Skåne, Sweden | 56°25’57”N; 12°34’18”E | TimA/TimB, S30/5FK, 4FB/1806R \| KC529512 | NA |
| *Vejdovskya ignava* Ax, 1951 | B | Hanko, Finland | 59°49’21”N; 22°58’21”E | TimA/TimB \| KC529513 | Neodal28SFa/Neodal28SRb (DK28S65) \| KC529635 |
| **Graffillidae Graff, 1908** |  |  |  |  |  |
| Graffillinae Graff, 1905 |  |  |  |  |  |
| *Graffilla buccinicola* Jameson, 1897 | MS | NA | NA | AJ012521 | NA |
| Pseudograffillinae Meixner, 1938 |  |  |  |  |  |
| *Pseudograffilla arenicola* Meixner, 1938 | BS | Törö, Stockholms Län, Sweden | 58°48’42”N; 17°47’42”E | TimA/TimB, S30/5FK, 4FB/1806R \| KC529514 | NA |
| Bresslauillinae Bresslau, 1933 |  |  |  |  |  |
| *Bresslauilla relicta* Reisinger, 1929 | M | Hanko, Finland | 59°49’50”N; 23°09’33”E | TimA/TimB \| KC529515 | LSU5/LSUD6.3 \| KC529636 |
| **Pterastericolidae Meixner, 1926** |  |  |  |  |  |
| *Pterastericola australis* Cannon, 1986 | MS | Australia | NA | AJ012518 | AY157161 |
| *Pterastericola psilastericola* (Jespersen and Lützen, 1972) Jondelius, 1992 | MS | Skagerrak, Sweden | 58°46’39”N; 10°41’55”E | – \| KC529516 | LSU5/LSUD6.3 \| KC529637 |
| **Umagillidae Wahl, 1910** |  |  |  |  |  |
| Umagillinae Wahl, 1910 |  |  |  |  |  |
| *Seritia elegans* (Westblad, 1953) Cannon, 1982 | MS | Korsfjorden, Bergen, Norway | 60°11’00”N; 05°12’15”E | TimA/TimB, S30/5FK, 4FB/1806R \| KC529517 | LSU5/LSUD6.3 \| KC529638 |
| *Wahlia macrostylifera* Westblad, 1930 | MS | Korsfjorden, Bergen, Norway | 60°11’00”N; 05°12’15”E | TimA/TimB, S30/5FK, 4FB/1806R \| KC529518 | LSU5/LSUD6.3 \| KC529639 |
| *Anoplodium stichopi* Bock, 1925 | MS | NA | NA | AF167424 | NA |
| **Solenopharyngidae Graff, 1882** |  |  |  |  |  |
| *Solenopharyngidae* sp. | M | Costa Paradiso, Sardinia, Italy | 41°03’09”N; 8°56’16”E | TimA/TimB (DK18S35cycli) \| KC529519 | SolenoF1/SolenoR  (DK28S68) \| KC529640 |
| Solenopharynginae Ehlers, 1972 |  |  |  |  |  |
| *Adenopharynx mitrabursalis* Ehlers, 1972 | M | Sylt, Germany | 55°02’06”N; 08°24’29”E | TimA/TimB \| KC529520 | LSU5/LSUD6.3B \| KC529641 |
| *Austradenopharynx* sp. | M | Poonindie, South Australia, Australia | 34°35’31”S; 135°54’12”E | TimA/TimB \| KC529521 | SolenoF1/SolenoR  (DK28S68) \| KC529642 |
| *Trisaccopharynx westbladi* Karling, 1940 | M | Kristineberg, Sweden | NA | AY775774 | NA |
| ***Incertae sedis*** |  |  |  |  |  |
| *Dalyellioida* “houdini” sp. | M | Darwin, Northern Territory, Australia | 12°26’06”S; 130°49’58”E | Neodal18SF/Neodal18SR (DK18S65) \| KC529522 | NA |
| *Dalyellioida* sp. | M | Costa Paradiso, Sardinia, Italy | 41°03’09”N; 8°56’16”E | TimA/TimB \| KC529523 | LSU5/LSUD6.3 \| KC529643 |
| *Neodalyellida* sp. 1 | M | Donaña NP, Andalusia, Spain | 36°52’16”N; 06°25’45”W | Neodal18SF/Neodal18SR (DK18S65) \| KC529524 | Neodal28SFa/Neodal28SRb (DK28S65) \| KC529644 |
| *Neodalyellida* sp. 2 | M | Doha, Qatar | 25°19’09”N; 51°32’16”E | Neodal18SF/Neodal18SR (DK18S65) \| KC529525 | Neodal28SFa/Neodal28SRb  (DK28S65) \| KC529645 |
| ***Outgroup*** |  |  |  |  |  |
| *Placorhynchus octaculeatus* Karling, 1931 | B | Hanko, Finland | 59°50’55”N; 23°15’20”E | TimA/TimB \| KC602395 | LSU5/LSUD6.3 \| KC602397 |
| *Acrorhynchides robustus* (Karling, 1931) Strand, 1928 | M | Sylt, Germany | 55°00’41”N; 08°25’30”E | TimA/TimB \| KC602396 | LSU5/LSUD6.3 \| KC602398 |

Additional sequences that were taken from GenBank are also listed with their GenBank accession number and when known, their sampling location. M: marine; F: freshwater; B: brackish water; L: limnoterrestrial; S: symbiotic.
