# Supplementary material for: A Comprehensive Molecular Phylogeny of Dalytyphloplanida (Platyhelminthes: Rhabdocoela) Reveals Multiple Escapes from the Marine Environment and Origins of Symbiotic Relationships
Source: PLoS One. 2013 Mar 25;8(3):e59917. doi: 10.1371/journal.pone.0059917 (PMC3607561; doi:10.1371/journal.pone.0059917)
Supplement: Table S2 — Identical sequences removed from the analyses. (DOC) [file pone.0059917.s004.doc]

| **Table S2.** Identical sequences removed from the analyses. | | | |
| --- | --- | --- | --- |
| **Sequences of taxa retained in the analyses** | **Taxa with identical sequences after processed alignment and removed from the analyses** | | |
|  | **18S+28S** | **18S** | **28S** |
| *Kytorhynchidarum* sp. 1 | *Kytorhynchidarum* sp. 2 | *Kytorhynchidarum* sp. | *Kytorhynchidarum* sp. 2 |
|  | *Kytorhynchidarum* sp. 3 | *Kytorhynchidarum* sp. 2 | *Kytorhynchidarum* sp. 3 |
|  |  | *Kytorhynchidarum* sp. 3 |  |
| *Gieysztoria cuspidata* (Canada) | *Gieysztoria cuspidata* (Belgium) | *Gieysztoria cuspidata* (Belgium) | *Gieysztoria cuspidata* (Belgium) |
| *Gieysztoria* “brown” n. sp. 6 |  |  | *Gieysztoria complicata* |
| *Microdalyellia fusca* |  | *Microdalyellia armigera* (Spain) |  |
|  |  | *Microdalyellia armigera* (Finland) |  |
| *Microdalyellia nanella* |  | *Microdalyellia rossi* |  |
|  |  | *Microdalyellia kupelwieseri* |  |
|  |  | *Microdalyellia fairchildi* (Finland) |  |
|  |  | *Microdalyellia picta* |  |
| *Castrada viridis* | *Castrada neocomensis* | *Castrada neocomensis* |  |
| **# identical sequences removed prior to analysis** | **4** | **11** | **4** |
