# Supplementary material for: A Comprehensive Molecular Phylogeny of Dalytyphloplanida (Platyhelminthes: Rhabdocoela) Reveals Multiple Escapes from the Marine Environment and Origins of Symbiotic Relationships
Source: PLoS One. 2013 Mar 25;8(3):e59917. doi: 10.1371/journal.pone.0059917 (PMC3607561; doi:10.1371/journal.pone.0059917)
Supplement: Table S3 — Test of substitution saturation with DAMBE v5.2.57 according to Xia's method for more than 32 OTUs. Analyses performed on all sites with gaps treated as unknown data. Subsets of 4, 8, 16 and 32 OTUs were randomly sampled 60 times and the test was performed for each subset. Iss: simple index of substitution saturation; Iss,cSym: critical Iss assuming a symmetrical topology; Iss,cAsym: critical Iss assuming an asymmetrical topology. If Iss is significantly smaller than Iss,c, little substitution saturation is present. Although Iss(18S+28S) does not significantly differ from Iss,cAsym(18S+28S), asymetrical trees are highly unlikely for our datasets. (DOC) [file pone.0059917.s005.doc]

| **Table S3.** Test of substitution saturation with DAMBE v5.2.57 according to Xia’s method for more than 32 OTUs. | | | | | |
| --- | --- | --- | --- | --- | --- |
| **NumOTO** | **Iss** | **Iss,cSym** | **P** | **Iss,cAsym** | **P** |
| **18S+28S** |  |  |  |  |  |
| 4 | 0.612 | 0.847 | 0.0000 | 0.830 | 0.0000 |
| 8 | 0.550 | 0.835 | 0.0000 | 0.747 | 0.0000 |
| 16 | 0.546 | 0.821 | 0.0000 | 0.654 | 0.0000 |
| 32 | 0.527 | 0.804 | 0.0000 | 0.541 | 0.1077 |
| **18S** |  |  |  |  |  |
| 4 | 0.279 | 0.835 | 0.0000 | 0.806 | 0.0000 |
| 8 | 0.254 | 0.812 | 0.0000 | 0.714 | 0.0000 |
| 16 | 0.252 | 0.795 | 0.0000 | 0.615 | 0.0000 |
| 32 | 0.257 | 0.778 | 0.0000 | 0.500 | 0.0000 |
| **28S** |  |  |  |  |  |
| 4 | 0.326 | 0.829 | 0.0000 | 0.797 | 0.0000 |
| 8 | 0.318 | 0.801 | 0.0000 | 0.698 | 0.0000 |
| 16 | 0.320 | 0.784 | 0.0000 | 0.595 | 0.0000 |
| 32 | 0.314 | 0.765 | 0.0000 | 0.473 | 0.0000 |

Analyses performed on all sites with gaps treated as unknown data. Subsets of 4, 8, 16 and 32 OTUs were randomly sampled 60 times and the test was performed for each subset. Iss: simple index of substitution saturation; Iss,cSym: critical Iss assuming a symmetrical topology; Iss,cAsym: critical Iss assuming an asymmetrical topology. If Iss is significantly smaller than Iss,c, little substitution saturation is present. Although Iss(18S+28S) does not significantly differ from Iss,cAsym(18S+28S), asymetrical trees are highly unlikely for our datasets.
